# Supplementary material for: The Newfoundland and Labrador mosaic founder population descends from an Irish and British diaspora from 300 years ago
Source: Commun Biol. 2023 Apr 28;6:469. doi: 10.1038/s42003-023-04844-9 (PMC10147672; doi:10.1038/s42003-023-04844-9)
Supplement: Supplementary file 1 — Supplementary Information [file 42003_2023_4844_MOESM1_ESM.pdf]

Supplementary Information for

The Newfoundland and Labrador mosaic founder population descends from an Irish and British diaspora from 300 years ago.

Authors: Edmund Gilbert[1,2], Heather Zurel[3], Margaret E. MacMillan[3], Sedat Demiriz[3], Sadra Mirhendi[3], Michael Merrigan[4], Seamus O'Reilly[4], Anne M. Molloy[5], Lawrence C. Brody[6], Walter Bodmer[7], Richard A. Leach[3], Roderick E. M. Scott[3], Gerald Mugford[3], Ranjit Randhawa[3], J. Claiborne Stephens[3], Alison L. Symington[3], Gianpiero L. Cavalleri[1,2], Michael S. Phillips[3].

Affiliations:

1. School of Pharmacy and Biomolecular Sciences, Royal College of Surgeons in Ireland, Dublin, Ireland.
2. FutureNeuro SFI Research Centre, Royal College of Surgeons in Ireland, Dublin, Ireland.
3. Sequence Bioinformatics, Inc., St. John's, Newfoundland, Canada.
4. Genealogical Society of Ireland, Dún Laoghaire, Ireland.
5. School of Medicine, Trinity College, Dublin, Ireland.
6. Genome Technology Branch, National Human Genome Research Institute, National Institutes of Health, Bethesda, MD, 20892, USA.
7. Weatherall Institute of Molecular Medicine, John Radcliffe Hospital, Oxford, United Kingdom.

*** Corresponding Author**: Edmund Gilbert

Email: edmundgilbert@rcsi.ie

Table of Contents

Supplemental Note 1 3
NF-Ancestry Identification 3
Supplemental Note 2 5
NL Geospatial Structure 5
Comparison to Zhai et al 9
NL Genetic Structure and Religious Identity 11
NL Indigenous Ancestry 14
NL Haplotype Structure 16
Supplemental Note 3 19
Estimated Effective Migration Surface Analysis 19
Supplemental Note 4 24
NL and Irish-British Structure 24
Supplemental Note 5 26
Relationship between British-Irish Ancestry and Religious Denomination 26
IBD Copying Profiles Across Ireland, Britain, and NL 28
Supplemental Note 6 32
Evidence of Irish-British Admixture in NL from fastGLOBETROTTER 32
fastGLOBETROTTER Coancestry Curves 34
Supplementary Note 7 41
Evaluating IBD Segment Calling 41
Supplemental Note 8 47
Supplementary References 49

Supplemental Note 1

NF-Ancestry Identification

To identify continental-scale ancestry within the NLGP 2,446 individuals we projected the genotypes of the NLGP individuals onto the 3,942 individuals from a combined dataset of individuals from the 1000 Genomes Project (1KGP)[1] and the Human Genome Diversity Project (HGDP)[2] – see Methods for details. Shown below are the principal components calculated from this analysis.

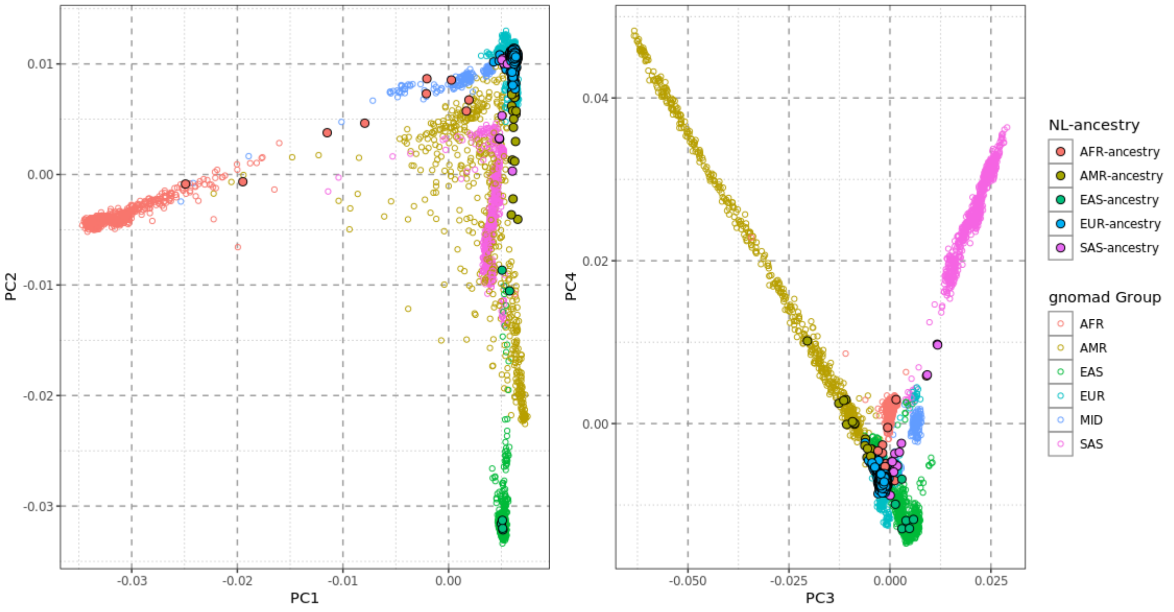

Supp Figure 1 – The first versus second and the third versus fourth principal components (PC) of NLGP individuals (circles with black borders), and ancestry references from either the HGDP or 1KGP datasets (hollow circles). Reference individuals are colour coded by ancestry groups defined by the gnomad dataset; AFR (African), AMR (American), EAS (East Asian), EUR (European), MID (Middle Eastern), SAS (South Asian) NLGP individuals are colour coded by inferred ancestry groups based on PC space.

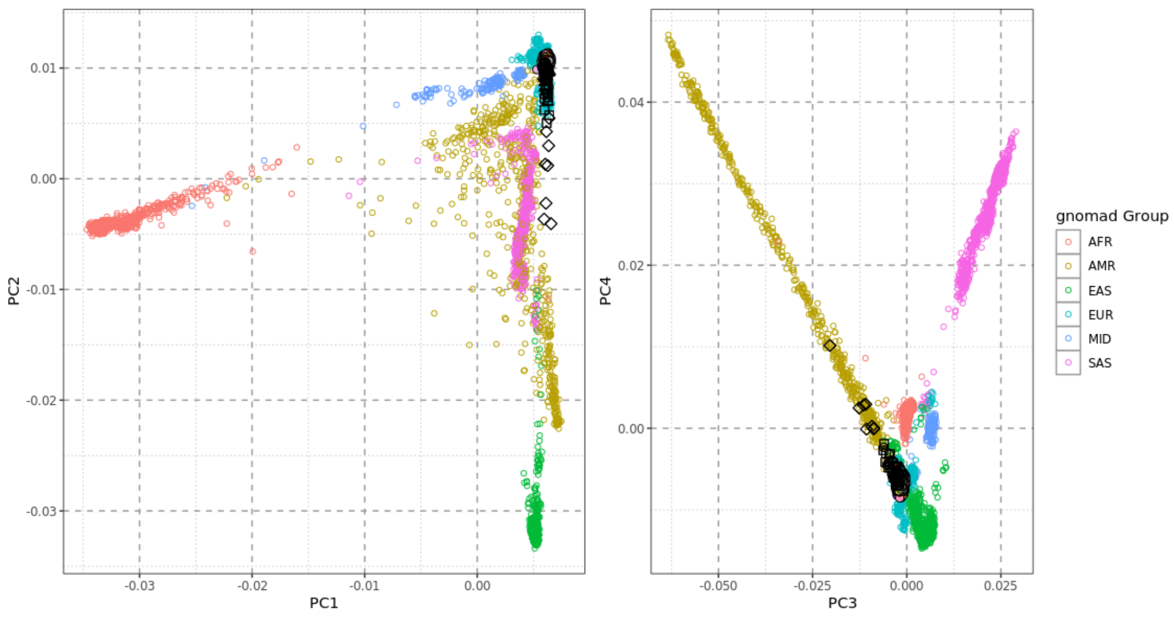

Supp Figure 2 – The first versus second and the third versus fourth principal components (PC) of ancestry references from either the HGDP or 1KGP datasets (hollow circles) with NLGP individuals projected onto the PC space calculated from references. NLGP individuals are only the 1,807 individuals studied as the NL-ancestry dataset, and colour and shape coded according to *fineSTRUCTURE* cluster. Khaki-colour clusters contain membership of individuals with inferred proportions of Indigenous ancestry.

Supplemental Note 2

NL Geospatial Structure

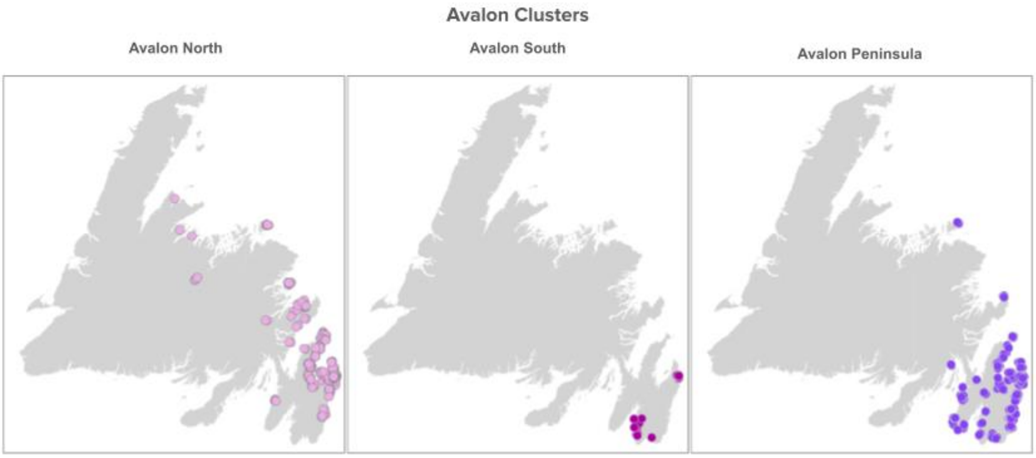

Supp Figure 3 – Geographic distribution of clusters with recent ancestors from the Avalon Peninsula. Shown below is the geographic distribution of individual NL *fineSTRUCTURE* clusters. Each point represents the birthplace of a grandparent of one of the $NL_{1,807}$ individuals, with a jitter introduced to aid visualisation.

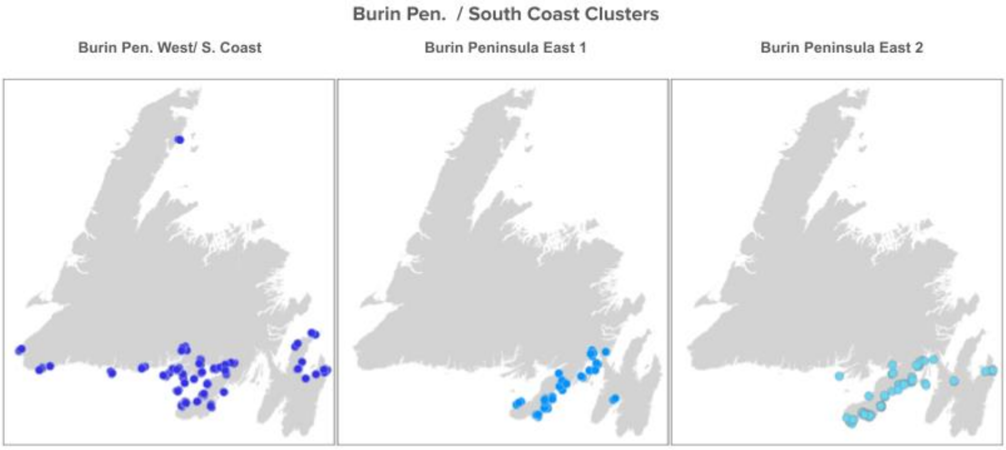

Supp Figure 4 – Geographic distribution of clusters with recent ancestors from the southern Newfoundland coast. Shown below is the geographic distribution of individual NL *fineSTRUCTURE* clusters. Each point represents the birthplace of a grandparent of one of the $NL_{1,807}$ individuals, with a jitter introduced to aid visualisation.

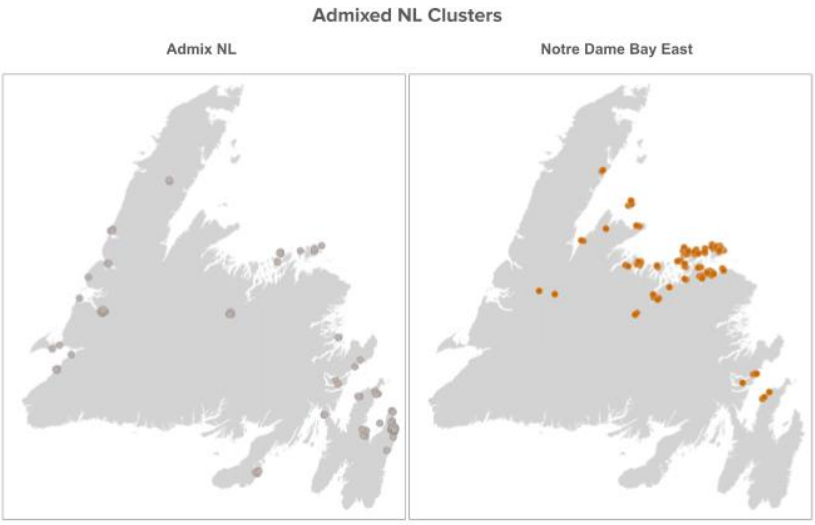

Supp Figure 5 – Geographic distribution of clusters of either mixed membership or geographic distribution around eastern Notre Dame Bay. Shown below is the geographic distribution of individual NL *fineSTRUCTURE* clusters. Each point represents the birthplace of a grandparent of one of the $NL_{1,807}$ individuals, with a jitter introduced to aid visualisation.

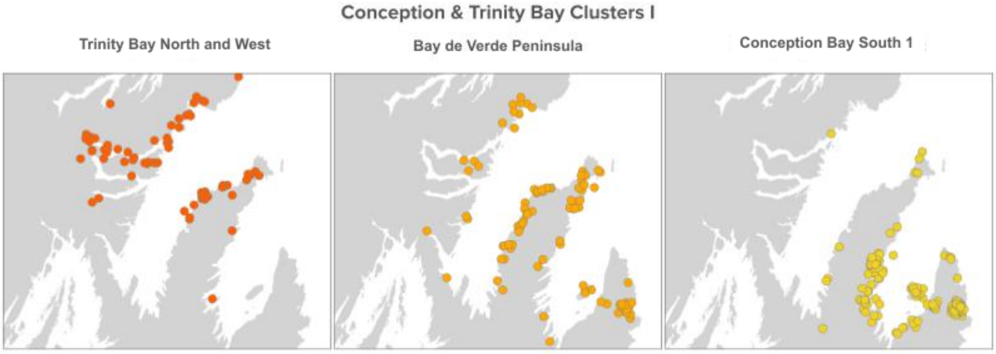

Supp Figure 6 – Geographic distribution of clusters predominantly located around Conception Bay. Shown below is the geographic distribution of individual NL *fineSTRUCTURE* clusters. Each point represents the birthplace of a grandparent of one of the $NL_{1,807}$ individuals, with a jitter introduced to aid visualisation.

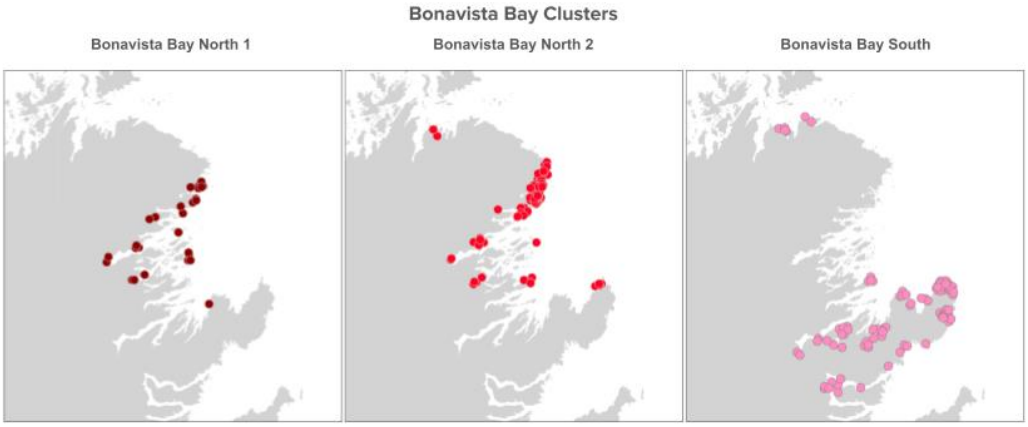

Supp Figure 7 – Geographic distribution of clusters predominantly located around Bonavista Bay. Shown below is the geographic distribution of individual NL *fineSTRUCTURE* clusters. Each point represents the birthplace of a grandparent of one of the $NL_{1,807}$ individuals, with a jitter introduced to aid visualisation.

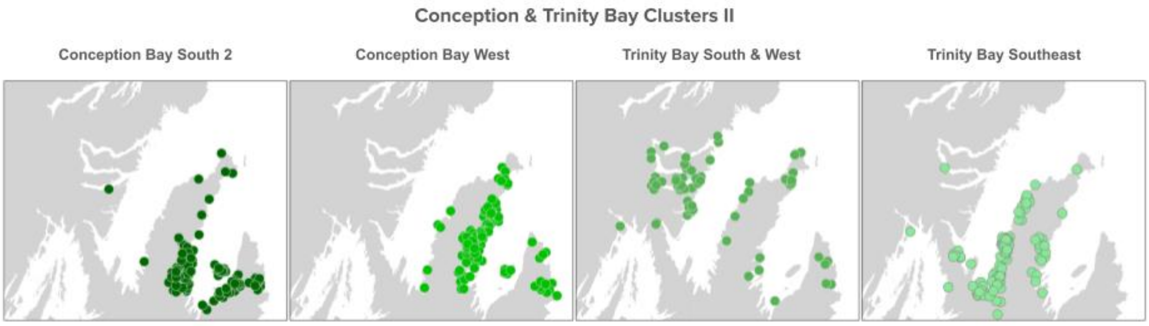

Supp Figure 8 – Geographic distribution of clusters predominantly located in either the Trinity or Conception Bays. Shown below is the geographic distribution of individual NL *fineSTRUCTURE* clusters. Each point represents the birthplace of a grandparent of one of the $NL_{1,807}$ individuals, with a jitter introduced to aid visualisation.

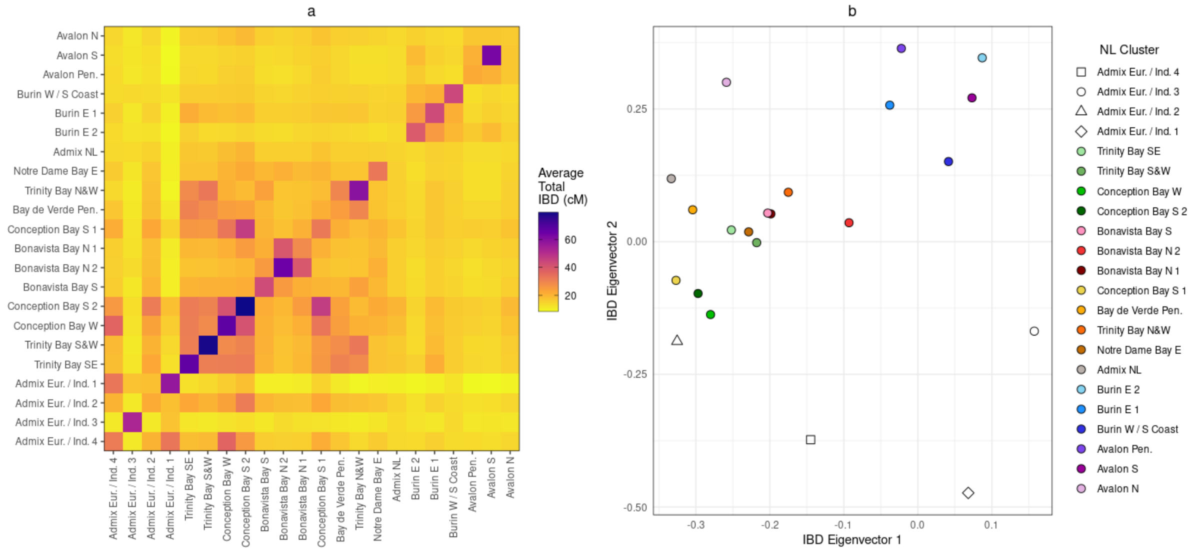

Supp Figure 8 - The degree of Identity-by-Descent (IBD) segment sharing between 22 *fineSTRUCTURE* clusters of 1,807 Newfoundland and Labrador individuals. (a) The sharing matrix of average per-individual-pair total sharing of IBD > 1 cM between and within NL clusters. Clusters are ordered according to the *fineSTRUCTURE* dendrogram. (b) The PCA of this sharing matrix, scaled and centred, showing the positions of each of the 22 clusters.

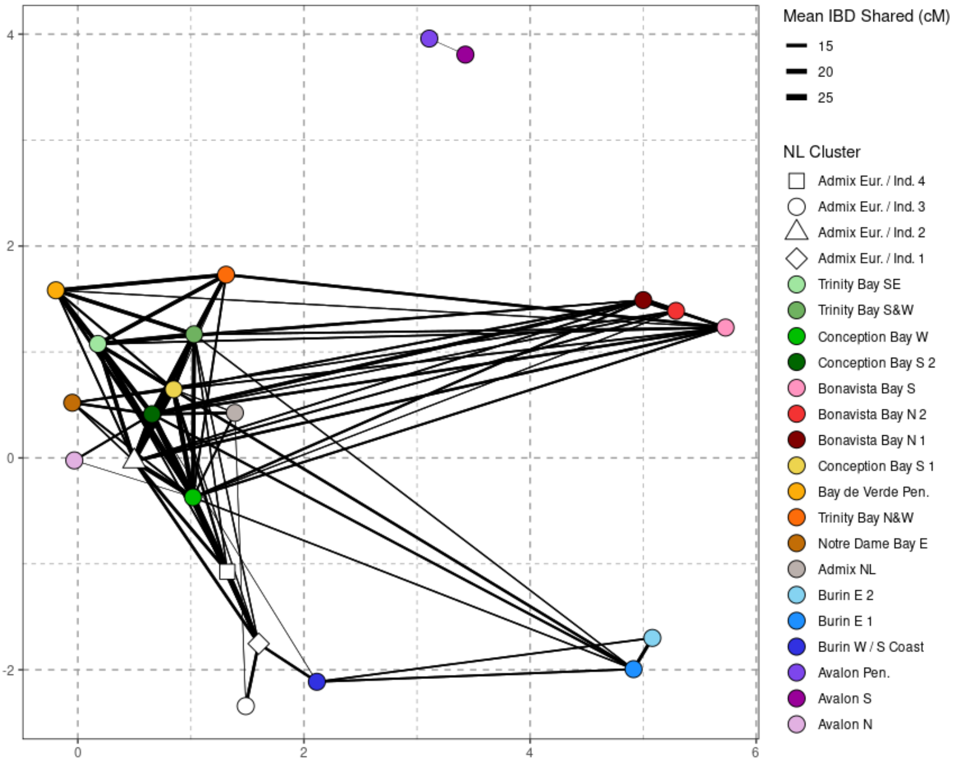

Supp Figure 9 - The Fruchterman-Reingold force projected layout of the network of IBD-sharing between the 22 NL *fineSTRUCTURE* clusters. IBD-sharing is the average total length of IBD > 3 cM and < 15 cM in length. To constructure the network, the minimum number of weight-ordered (descending) edges (IBD connections) which connected every cluster to at least one other cluster was used. Thus, a network of the largest 164 IBD-connections was used in R[3] using the igraph package[4]. This projection of the network was plotted using the R Package ggplot2[5]. Each node is one cluster, with the edge connections representing the mea average IBD-sharing between two clusters. This edge's thickness is proportional to the amount shared.

Comparison to Zhai et al

We directly compared our sample of the NL population with a previous, smaller, sample of 442 references reported by Zhai et al[6]. Using the Gene Expression Omnibus (GEO) accession GSE74392, we downloaded SNP-microarray genotype data generated on the Affymetrix Axiom Genome-Wide Array platform and merged with the genotype data from the NL Genome Project (NLGP) based on SNPs with the same variant rs identifier.

This common dataset included all individuals from the 1,807 dataset of NL-ancestry from the NLGP and 442 NL references from Zhai et al (henceforth the "Zhai" dataset), over 168,075 common SNPs. The substantially reduced marker set in this comparison precluded incorporation of the Zhai dataset into the main analyses of the NLGP dataset - though did allow the direct comparison of the $NL_{1,807}$ and Zhai datasets.

After standard quality control filtering of sample and marker missingness, minor-allele-frequency, and marker deviation from Hardy-Weinberg-Equilibrium expectations using the same thresholds in the $NL_{1,807}$ analysis (see Methods) this left 2,249 individuals and 167,968 common markers. We first investigated relatedness across the two datasets and found evidence that either the two datasets have sampled the same individual or the corresponding monozygotic twin 4 times, parent-offspring pairs 7 times, and siblings 3 times (Supp Figure 10).

We next compared the population genetics of each dataset to confirm that the NLGP is an equivalent sample of NL with regards to genetic variation. We performed PCA using the implementation within PLINK[7,8], pruning markers for linkage disequilibrium to gain an unlinked marker-set - using the PLINK command *--indep-pairwise 1000 50 0.2*. We further detected ROH segments using the PLINK *--homozyg* implementation with the following command: *--homozyg --homozyg-window-snp 50 --homozyg-snp 50 --homozyg-kb 1500 --homozyg-gap 1000 --homozyg-density 50 --homozyg-window-missing 5 --homozyg-window-het 1.*

We find that our NLGP sample of NL genetic ancestry matches well with Zhai dataset in principal component space (Supp Figure 11). Individuals from both datasets overlap over the three axis of variation over principal components 1 and 2, and in the space defined by principal components 3 and 4 we identify individuals from the Zhai dataset which overlap with individuals with putative Indigenous American ancestry from the NLGP dataset - matching observations made by Zhai et al[6] that they observed individuals with "aboriginal ancestry". We tested statistical evidence of difference between each dataset along any of the four principal components with Mann-Whitney test in R[3] with the *wilcox.test()* function, and found no evidence of difference along principal component 1 (p-value = 0.106), 2 (p-value = 0.4026), or 3 (p-value = 0.1444). We find evidence of difference along principal component 4 (p-value = 0.000299), though this may be due to the small sample sizes of individuals with Indigenous ancestry in either dataset. Lastly, we compared the profile of Runs of Homozygosity (ROH) between each of our $NL_{1,807}$ *fineSTRUCTURE* clusters and the Zhai dataset (Supp Figure 12) and found a general distribution of ROH in the Zhai sample similar to the distribution along the NLGP clusters.

These results provide demonstrable evidence that our wider results from analysis of the population genetics of Newfoundland and Labrador agree well with, and substantially expand, previous samples and investigation of this population.

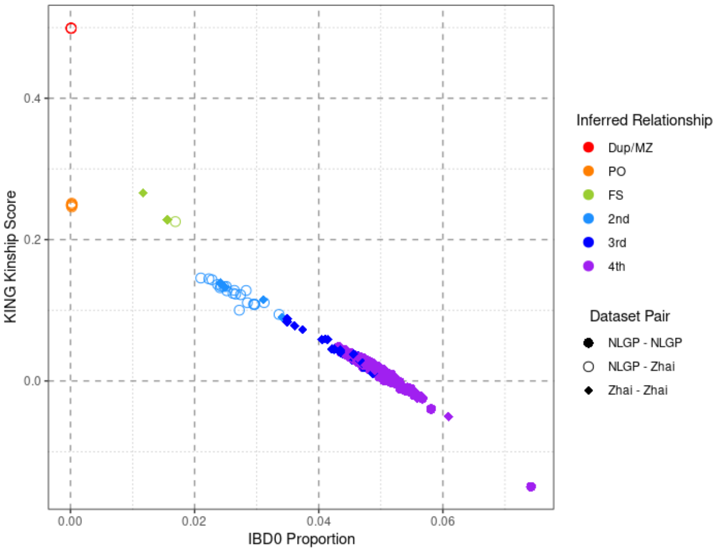

Supp Figure 10 - Relatedness across the NLGP and the Zhai datasets of Newfoundland and Labrador ancestry. Each point is a pair of related individuals, with colour indicating the type of relationship. Dup/MZ is either monozygotic twin or duplicate sample, PO stands for parent -offspring, FS for full-sibling, and 2nd-4th the degree of relationship beyond. The shape of the point indicates if the relationship is within or between the two NL-ancestry cohorts.

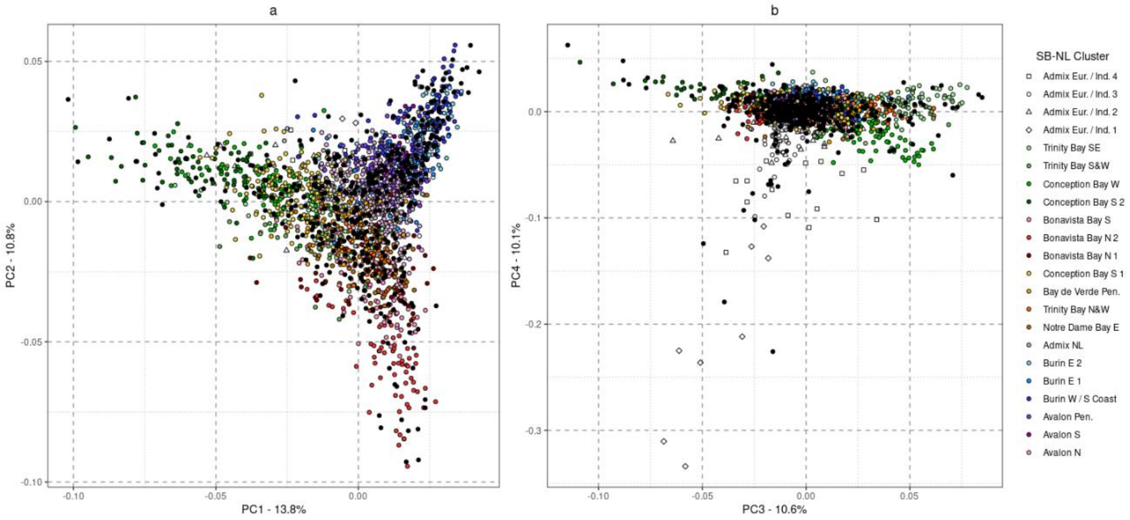

Supp Figure 11 - Shared genetic structure across the NL Genome Project (NLGP) and the Zhai dataset. The first four principal components estimated from PLINK, showing one versus two (a) and three versus four (b). Individuals from the NLGP are colour coded according to *fineSTRUCTURE* cluster memsbership, and Zhai individuals are shown as solid black point layered above. Each axis is labelled with the variance explained by that component estimated from the eigenvalues from PLINK.

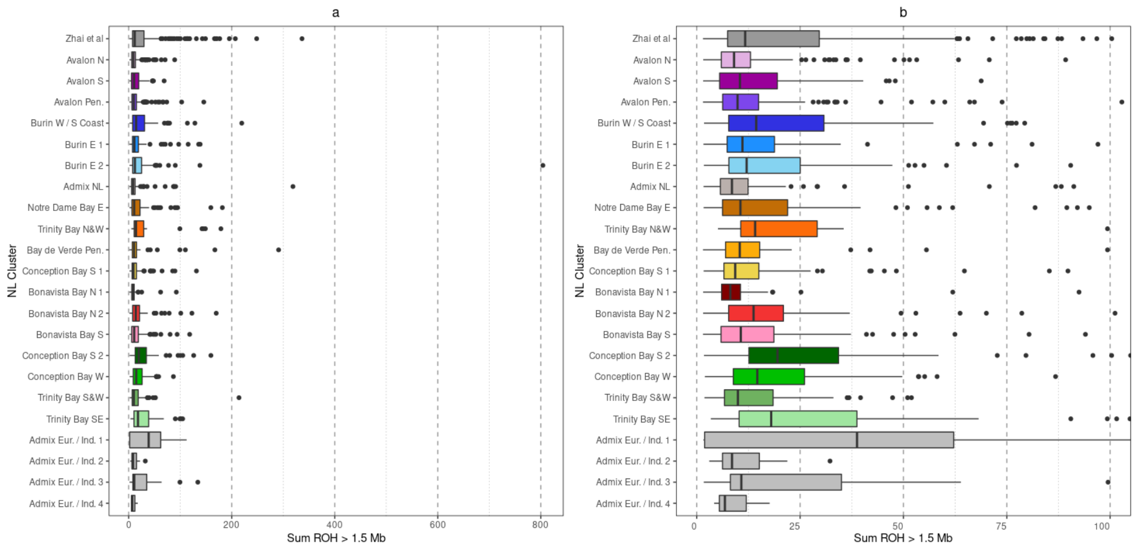

Supp Figure 12 - Distribution of Runs of Homozygosity across the NL Genome Project (NLGP) and the Zhai dataset. (a) The sum of ROH > 1.5 Mb in length detected by PLINK in NL-ancestry individuals across the 22 *fineSTUCTURE* clusters detected in the $NL_{1,807}$ dataset versus the Zhai dataset. (b) The ROH data shown in panel a but with axis limits of 100 Mb to show the main distribution of sum ROH across clusters. Boxplots show the median value, with lower and upper hinges showing the 1st and 3rd quartiles. Whiskers show the largest value no further than 1.5 x the Interquartile Range (IQR) from that range. Data points beyond these whiskeys are plotted separately as black points.

NL Genetic Structure and Religious Identity

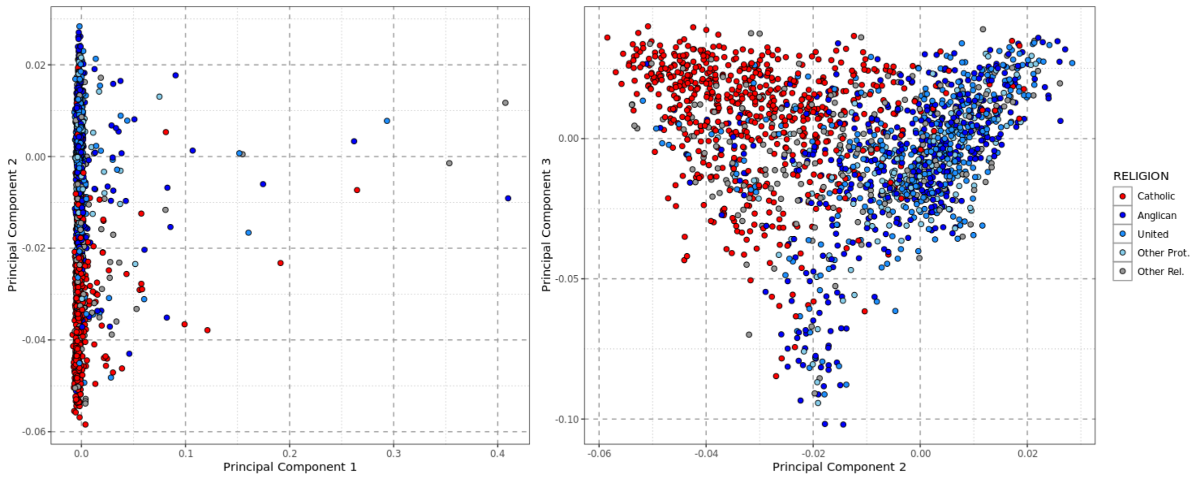

Supp Figure 13 – The first, second, and third principal components calculated from the "chunkcounts" coancestry matrix of the $NL_{1,807}$ individuals. Each point represents the position of one NL individual projected onto the genetic space – colour coded by religious background; with red indicating Catholic, blue indicating Protestant denominations, and grey indicating any other religious background.

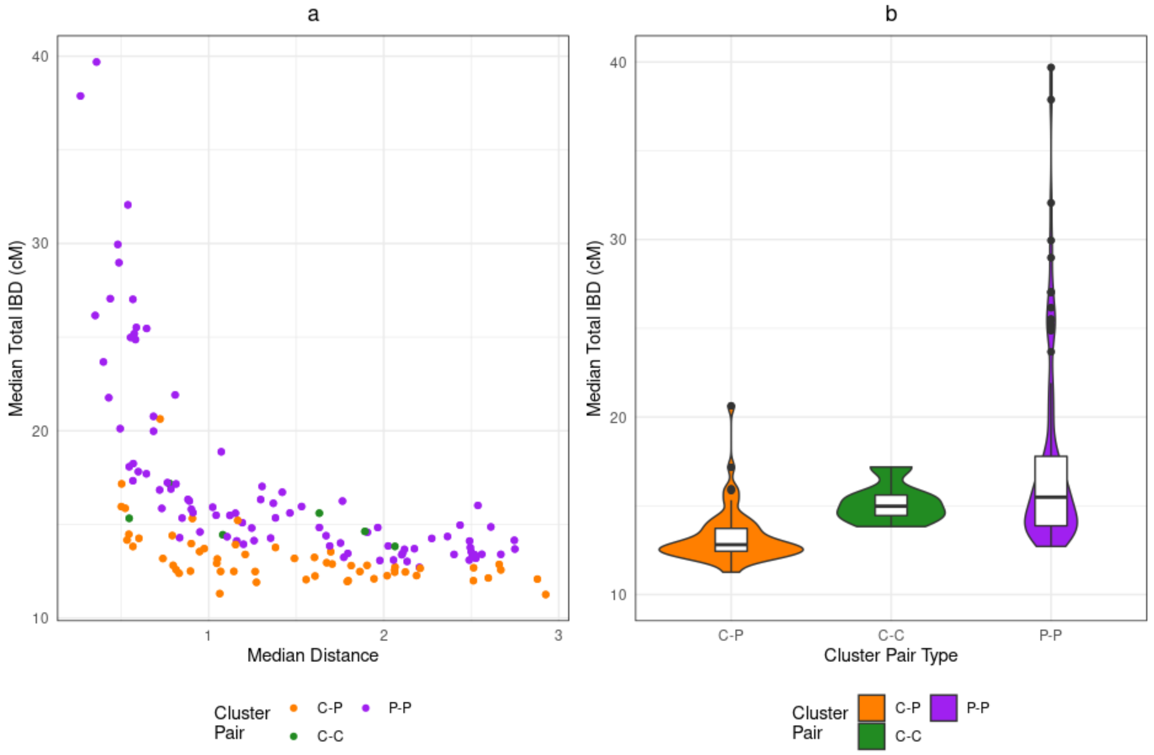

Supp Figure 14 - Relationship between geography and religion between NL *fineSTRUCTURE* clusters. (a) The median total length of IBD > 1 cM between a pair of NL clusters versus the median distance of an individuals' grandparents between the two clusters, where the value of each pair of clusters is shown as a point colour coded to whether it is a cluster of predominantly Protestant (P) membership or Catholic (C). (b) The distribution of median total lengths of IBD > 1 cM in the three types of cluster pairs; either Catholic-Protestant (C-P), Catholic-Catholic (C-C), or Protestant-Protestant (P-P). Boxplots show the median value, with lower and upper hinges showing the 1st and 3rd quartiles. Whiskers show the largest value no further than 1.5 x the Interquartile Range (IQR) from that range. Data points beyond these whiskeys are plotted separately as black points.

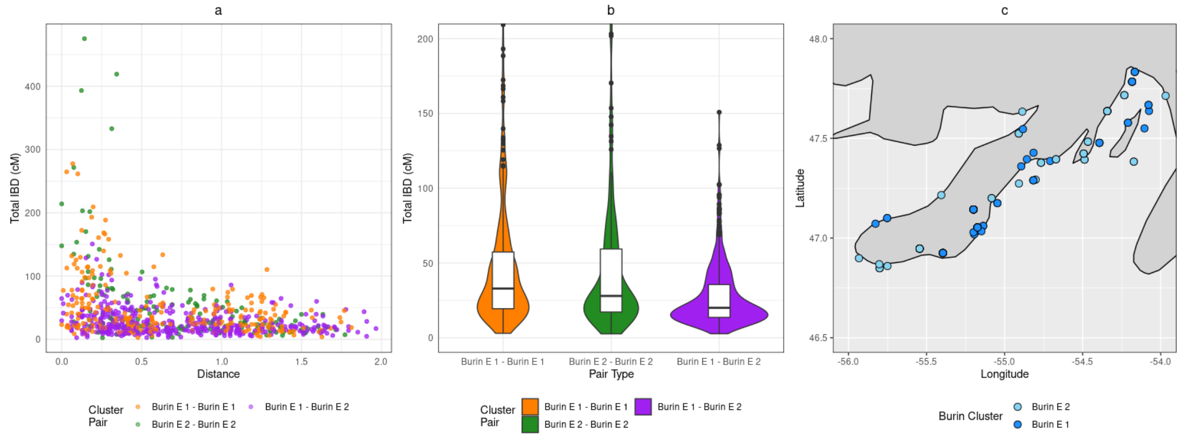

Supp Figure 15 - The fine-scale relationship between geography and genetic clusters in the Burin peninsula in the south coast of Newfoundland. (a) The total length of IBD > 1 cM between a pair of NL individuals with membership in either the *Burin E 1* or *Burin E 2 fineSTRUCTURE* clusters, versus the distance of an individuals' grandparents between the two clusters. Each pair of individuals are shown

as a point colour coded to which pair of clusters are represented. (b) The distribution of median total lengths of IBD > 1 cM in the three types of cluster pairs. (c) The geographic positions of the Burin peninsula grandparents used in this analysis. Boxplots show the median value, with lower and upper hinges showing the 1st and 3rd quartiles. Whiskers show the largest value no further than 1.5 x the Interquartile Range (IQR) from that range. Data points beyond these whiskeys are plotted separately as black points. Not in (b) we have limited the y-axis from ~450 cM to 200 cM to aid legibility and inter-group comparability.

NL Indigenous Ancestry

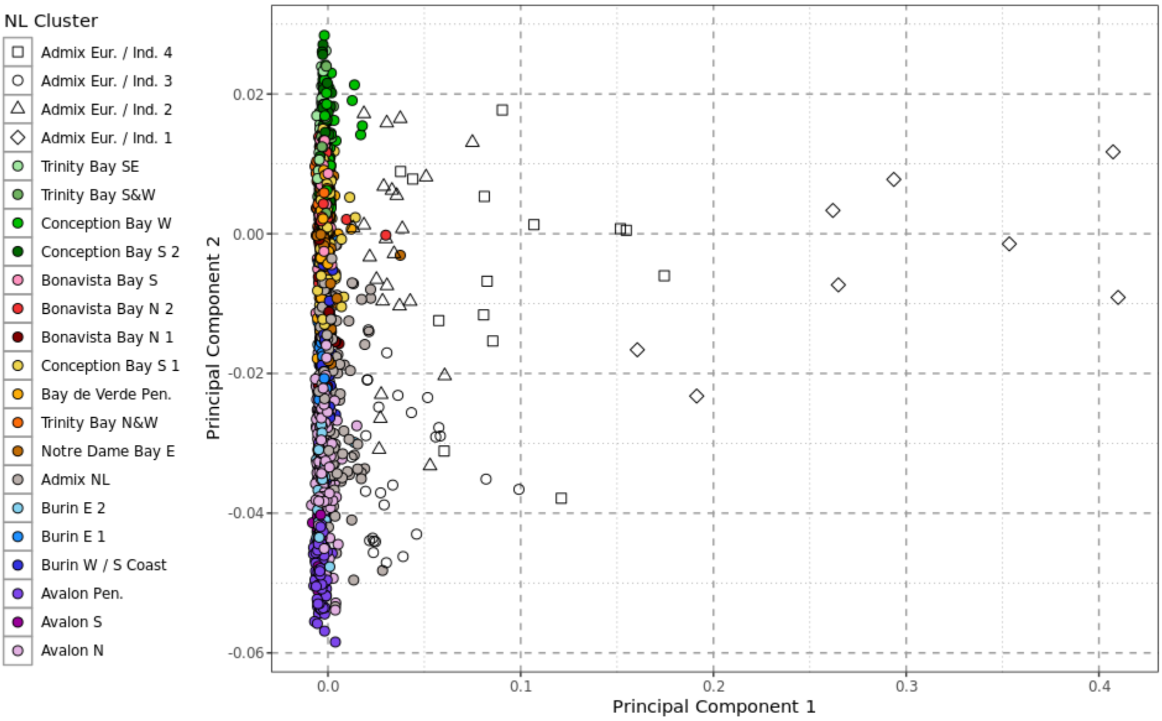

Supp Figure 16 – The first and second principal components calculated from PCA of *ChromoPainter* co-ancestry matrix of 1,807 NL ancestry individuals. Individual genotypes are represented by single points, which are shape and colour coded according to *fineStructure* clustering.

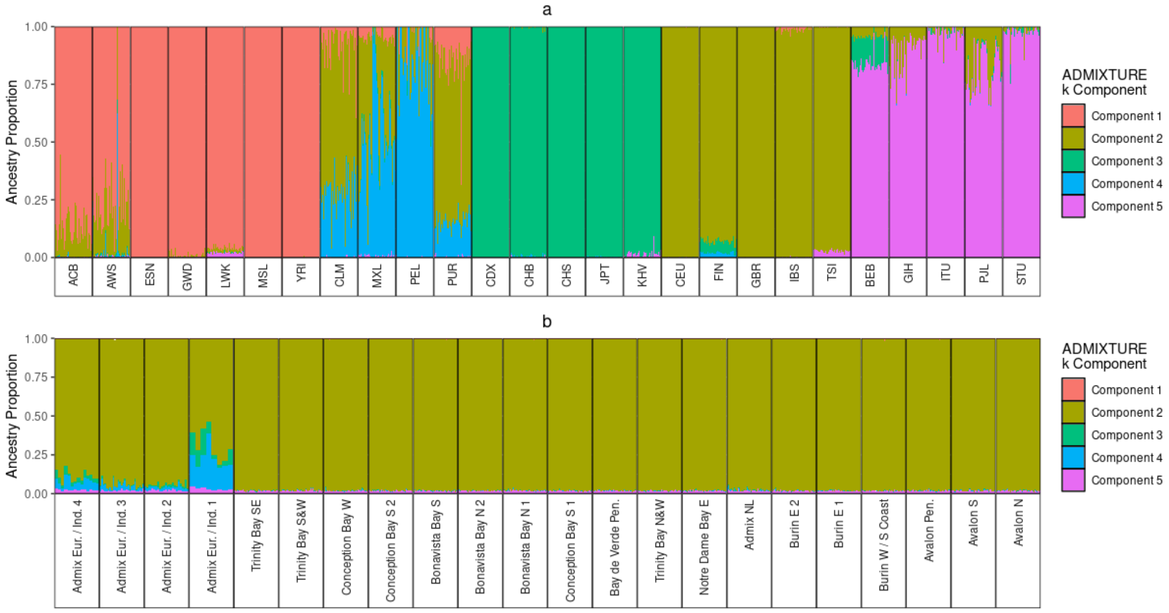

Supp Figure 17 – (A) ADMIXTURE[9] analysis with *k*=5 ancestry components calculated using allele frequencies of 1000 Genomes Phase 3[10] individuals, grouped by population and continental meta-group. The five ancestry components are given labels reflective of the continental group that component is maximised in. (B) Supervised ADMIXTURE analysis of 1,807 NL individuals grouped

according to fineSTRUCTURE[11] cluster, where the *k*=5 ancestry components estimated using 1000 Genomes references in A are estimated in the NL individuals.

NL Haplotype Structure

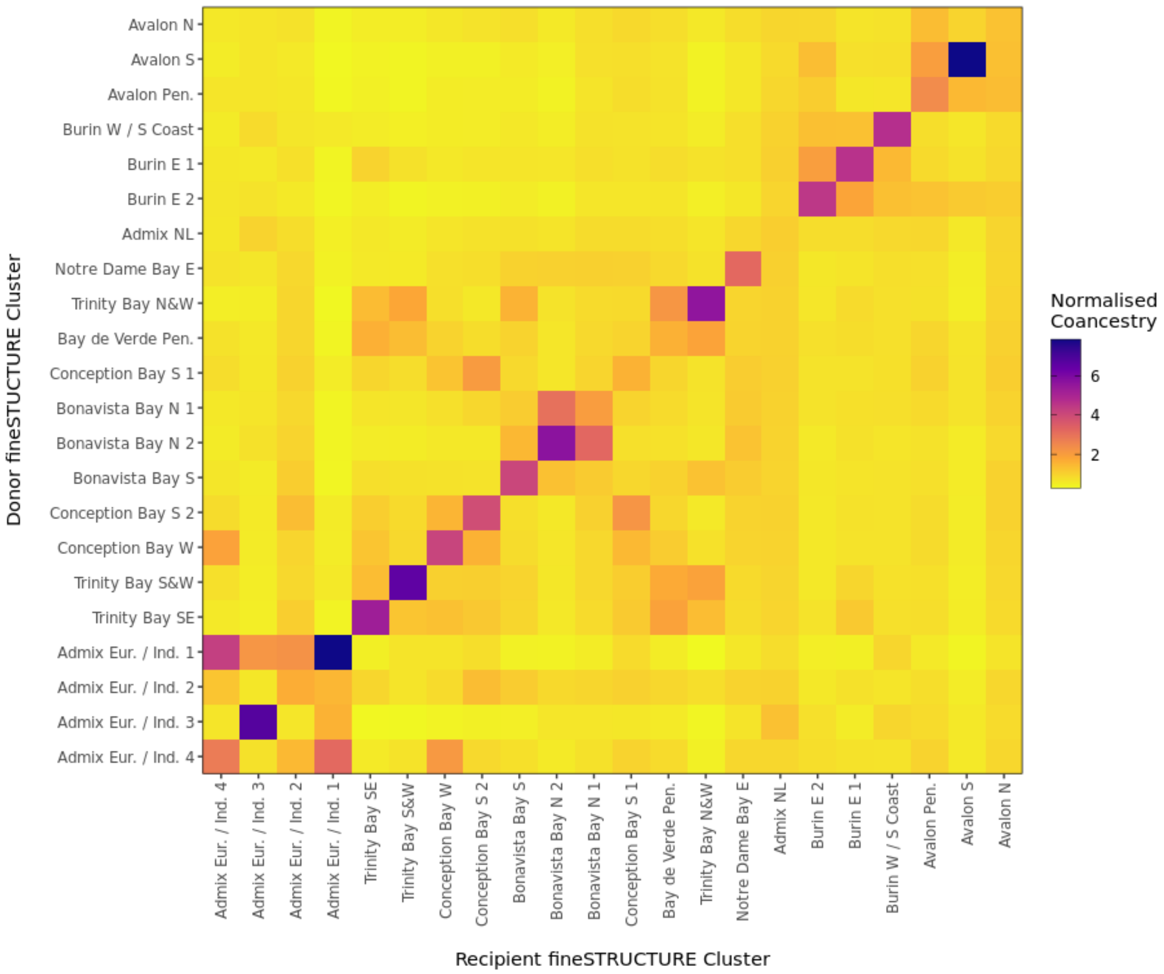

Supp Figure 18 – The column-normalised, cluster-averaged “chunklengths” coancestry matrix showing haplotype sharing between the 22 *fineSTRUCTURE* NL clusters.

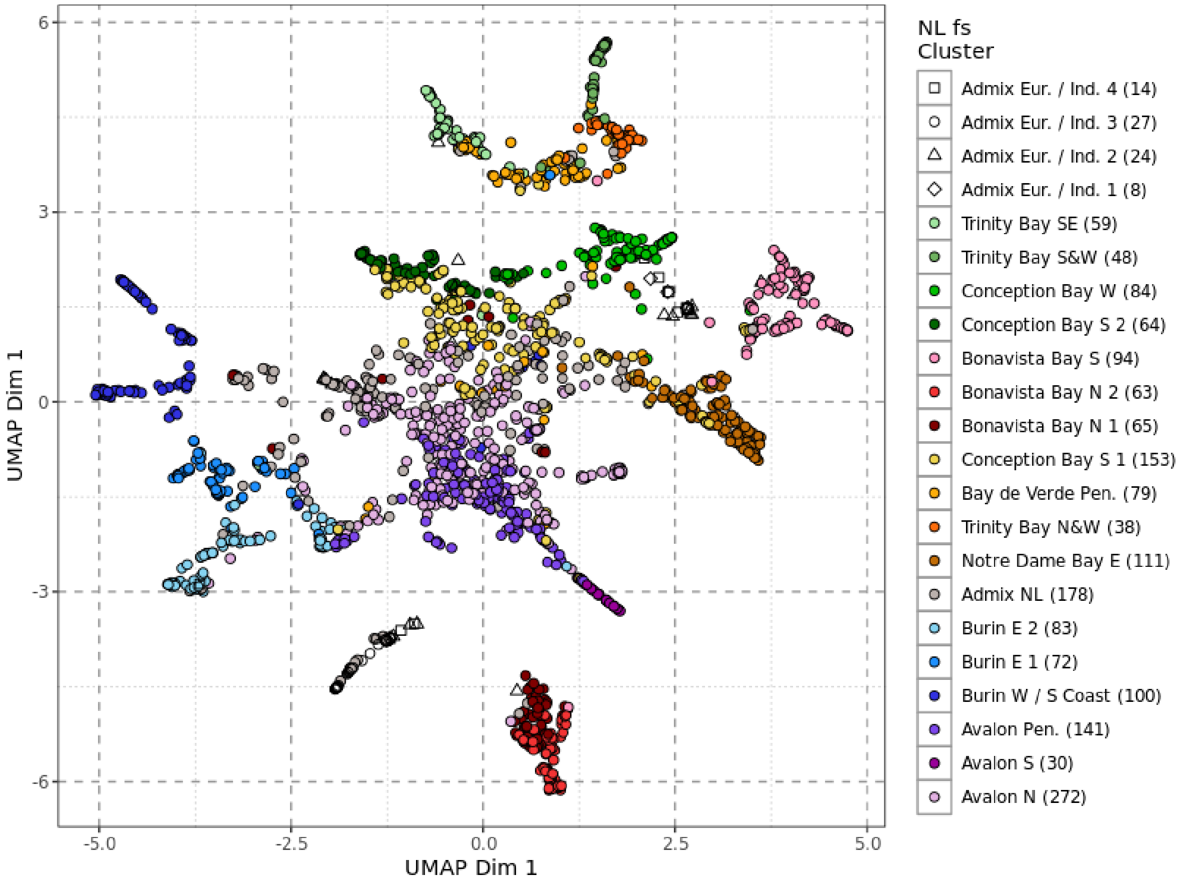

Supp Figure 19 – The umap[12] projection of the first 20 principal components calculated from the "chunkcounts" coancestry matrix using the R function umap from the uwot package using default parameters. Individual points represent the genetic coordinates of one individual, colour and shape coded according to *fineSTRUCTURE* cluster.

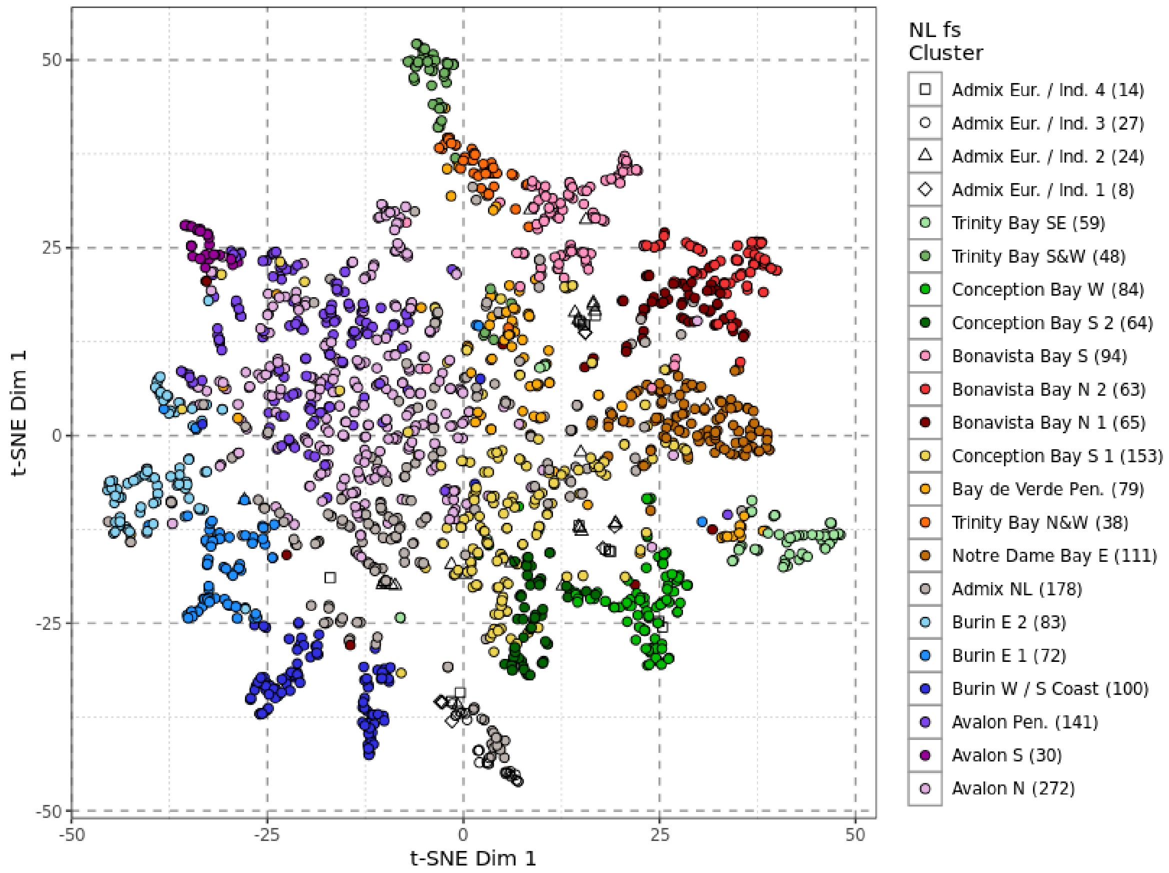

Supp Figure 20 – The t-SNE[13] projection of the first 20 principal components calculated from the "chunkcounts" coancestry matrix using the R function Rtsne from the Rtsne package using default parameters. Individual points represent the genetic coordinates of one individual, colour and shape coded according to *fineSTRUCTURE* cluster.

Supplemental Note 3

Estimated Effective Migration Surface Analysis

To further characterise the extent of structure in NL using the NLGP dataset, we applied the Estimated Effective Migration Surfaces (EEMS) software[14] to the genetic and geographic data from the $NLGP_{1,807}$ dataset. This would allow us to estimate a "migration surface", thereby revealing any discontinuity between genetic distance and geographic distance of samples grouped into the same geographic region - detecting gene flow corridors (that is more genetic similarity than expected given geographic distance), and gene flow barrier (that is less genetic similarity given geographic distance). This method has been used successfully in studies of Irish[15] and British[16] regions as well as the isolated Miyako Islands of the Ryukyu Archipelago, south of Japan[17].

We selected individuals with grandparental place-of-birth information from the 1,807 individuals in the NLGP dataset identified as "NL ancestry" (see main Results), selecting individuals with grandparents born within a close distance of each other. We selected these by calculating the distance between each grandparent for each NL individual (measured with latitude and longitude) and filtered for individuals with an average distance ≤ 0.5. From a total of 1,807 individuals with grandparental data, this left 739 individuals with genetic data and a geographic position which was calculated as the mean grandparent birthplace. Extracting genotypes from the 685,221 SNPs from the cleaned and filtered SNP-set (see Methods), we converted this genetic data into a matrix of genetic dissimilarities using the *bed2diffs* program included in the EEMS software download. To generate the outer boundaries of the analysed area, we utilised an online Google Maps API tool.

With this genetic and geographic data, we performed an initial EEMS analysis whereby we generated 10 initial replicate runs of the EEMS algorithm. We selected the replicate with the highest log-likelihood as a starting point for a final, "extension", EEMS run which would take the average of a further 10 replicate EEMS runs as the result, see recommended by the authors[14]. Each of the initial replicate runs had a different random seed, assuming 300 demes, with 5 million iterations of the EEMS MCMC algorithm, with the first 3 million used as burnin iterations, sampling every 10,000th iteration. We selected the fifth initial replicate based on the log-likelihood trace (Supp Figure 18).

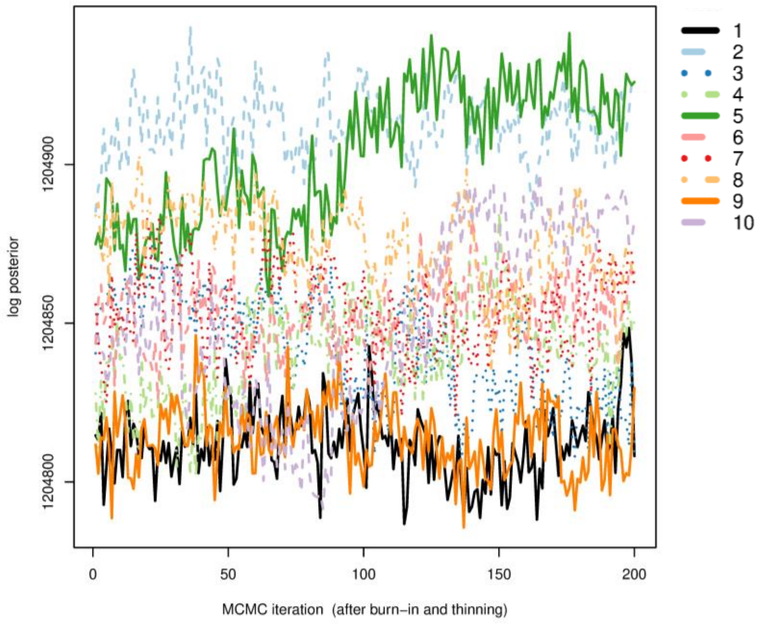

Supp Figure 21 - The log-likelihood trace of 10 initial replicate runs of the EEMS[14] algorithm on 739 NL individuals with genetic and grandparental geographic data. The fifth replicate run was chosen as at the end of the analysis it had the highest log-likelihood. Panel was plotted in the statistical computing language R[3] using the package *rEEMSplots*.

With this initial EEMS replicate, we extended the MCMC algorithm for a further 3 million MCMC iterations, using the first 2 million as additional burnin iterations, and sampling every 10,000th iteration, and assuming 300 demes. We processed this EEMS output using the R[3] package *rEEMSplots* which is also provided in the EEMS software download. All plots were generated using this package in R. We show the observed versus fitted dissimilarity between EEMS demes (Supp Figure 19), the log-likelihood trace of the 10-replicate extension runs (Supp Figure 20), and the estimated effective migration surface for our sample of NL (Supp Figure 21). Across most bays where sampling is concentrated the EEMS algorithm estimate a general gene flow barrier, which would be consistent with fine-scale structure beyond the resolution of the 300-deme model. Higher demes are possible but are computationally intensive due to the bioinformatic bottleneck of the MCMC iterations. Nevertheless, we do see some regions of gene flow within NL, notably at the bottom of the Avalon peninsula, which would be consistent with the Catholic-membership *fineSTRUCTURE*[11] clustering detected there (Figure 1 and 2), and the shared common Irish ancestry (Figure 3 and 5). Likewise, along the south coast of NL we detect a wide corridor, linking small demes together. This may be due to shared genetic ancestry (supported by the *fineSTRUCTURE* cluster *Burin W/S Coast*), and possible due to low sampling size on the south coast compared to eastern bays. A similar gene flow corridor, that is linking regions with similar *fineSTRUCTURE* clustering membership and low sampling density, joins occupied demes on the western coast with demes towards the centre of NL, where habitation is sparse[18]. We observe a strong region of gene flow barrier in the north-west of the island, seemingly centred around one small deme. *fineSTRUCTURE* clustering of these individuals shows this region sparsely populated with grandparents seemingly sampled from individual settlements (Figure 2). The grandparental sampling site which corresponds to the strong gene flow barrier detected includes grandparents of samples assigned to the *Trinity Bay N&W fineSTRUCTURE* cluster, where the neighbour sample sites are predominantly *Admix NL* and *Burin W/S Coast*. This discrepancy of clustering from neighbouring sites may explain this strong barrier in an otherwise unpopulated region of NL.

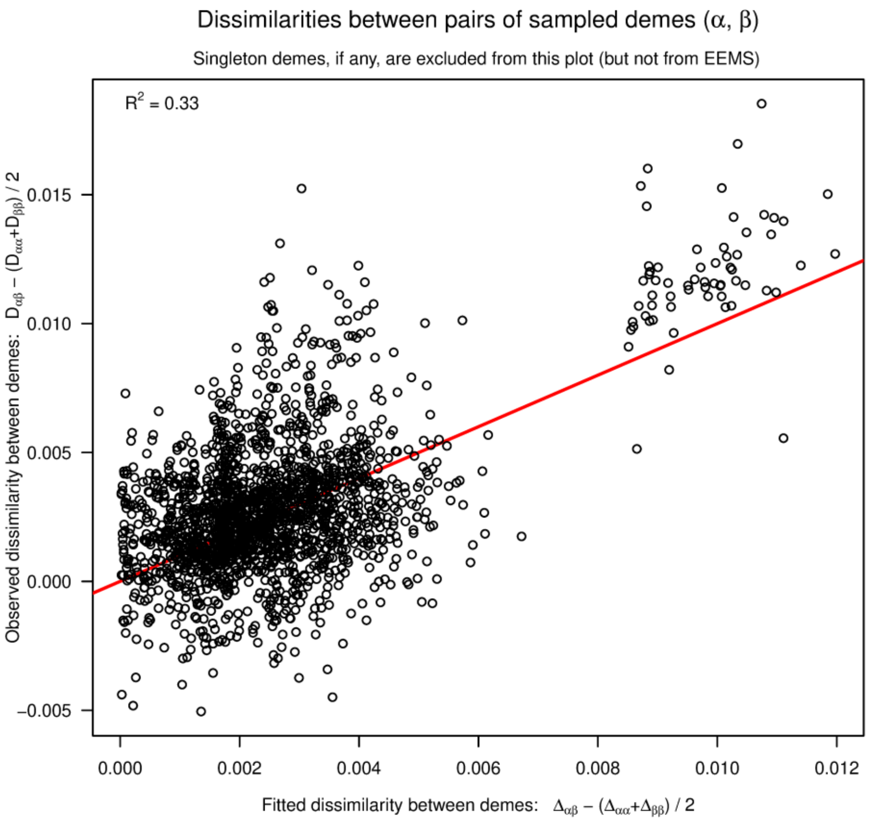

Supp Figure 22 - The scatter plot of fitted versus observed dissimilarities between demes in the final EEMS analysis. The trendline measured by the R^2 value (0.33) is shown in red. Each point represents a pair of sampled demes. Panel was plotted in the statistical computing language R[3] using the package *rEEMSplots*.

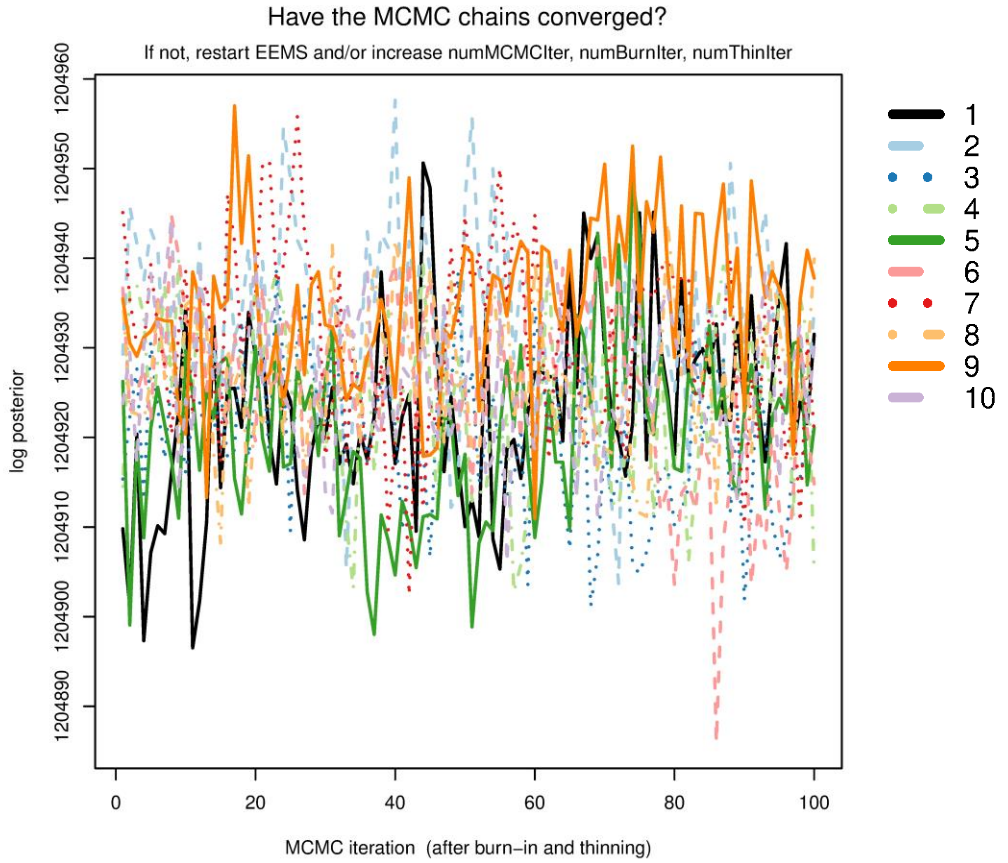

Supp Figure 23 - The log-likelihood trace of 10 extension replicate runs of the EEMS[14] algorithm on 739 NL individuals with genetic and grandparental geographic data. Panel was plotted in the statistical computing language R[3] using the package *rEEMSplots*.

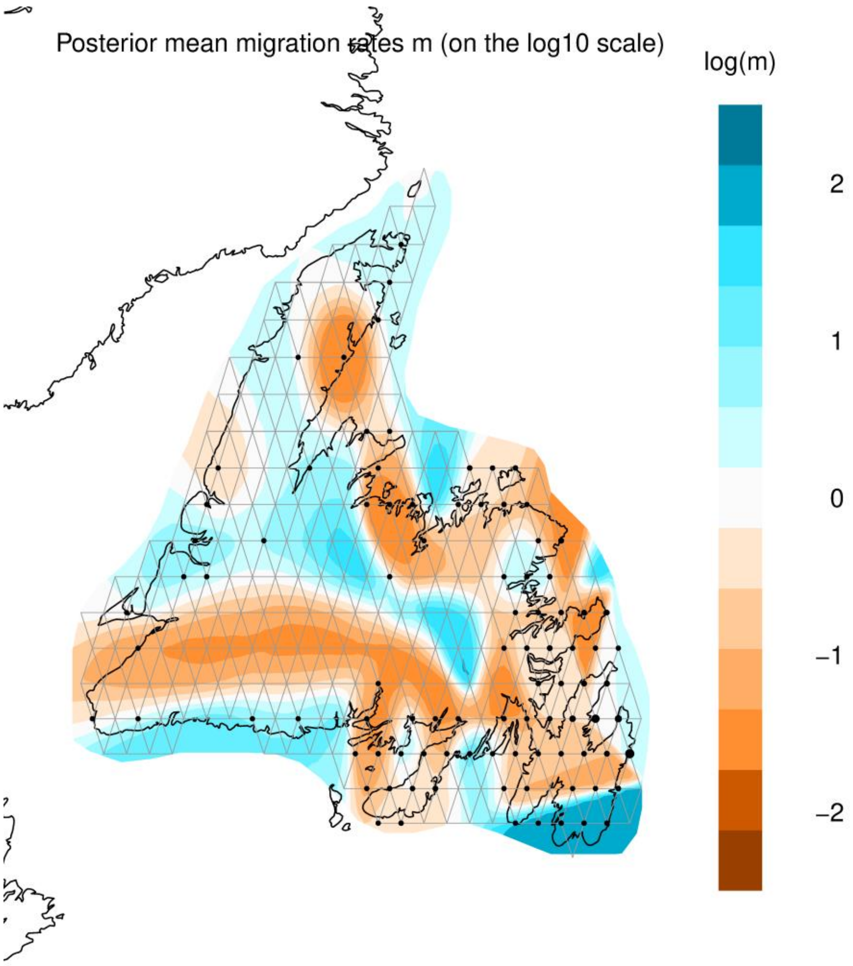

Supp Figure 24 - The estimated effective migration surface of the island of Newfoundland, using 739 individuals with placed at the average location of their grandparents. Each individual was assigned to one of a possible 300 demes, occupied demes shown with black circles. Migration rates were estimated along edges joining these demes, with the surface estimate shown in blue/red colouring, with lower expected gene flow shown in red, and higher, blue. Panel was plotted in the statistical computing language R[3] using the package *rEEMSplots*.

Supplemental Note 4

NL and Irish-British Structure

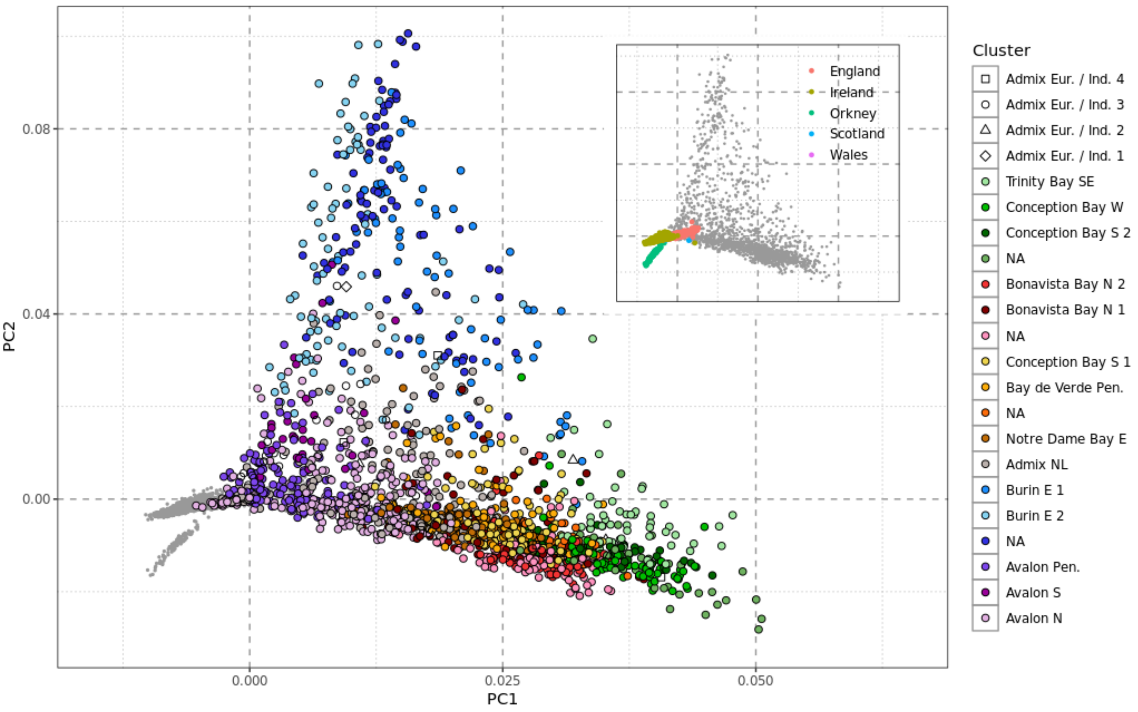

Supp Figure 25 – Principal Component 1 and 2 calculated from co-ancestry matrix of 1,807 NL individuals and 4,469 Irish-British references individuals. NL individuals are colour coded according to *fineSTRUCTURE* cluster membership, Irish or British references are shown as small grey circles with Orkney forming its own cloud. Irish or British individual positions alone are shown as an insert, with yellow indicating Irish, red English, blue Scottish, green Orcadian, and purple Walsh label. The rest of Ireland and Britain forms a cline along principal component 1, with Irish to English as component one increases.

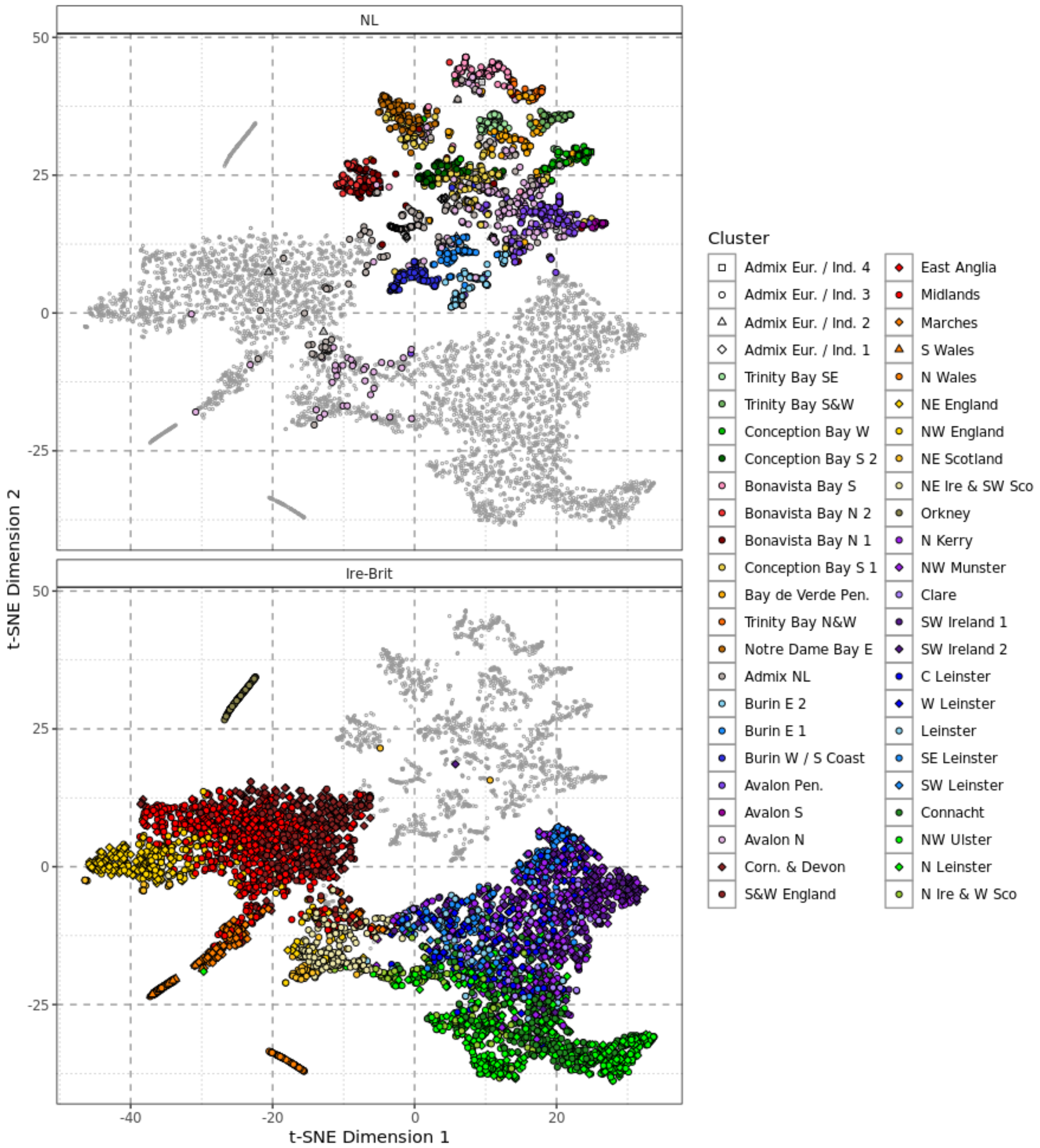

Supp Figure 26 – Genetic coordinates calculated from applying t-SNE to the first 20 principal components calculated from the coancestry matrix of 1,807 NL individuals and 4,469 Irish-British reference individuals. NL and Irish-British references are shown in separate panels, with colour and shape coding indicating *fineSTRUCTURE* (for NL individuals) or IBD-based clusters (for Irish-British individuals).

Supplemental Note 5

Relationship between British-Irish Ancestry and Religious Denomination

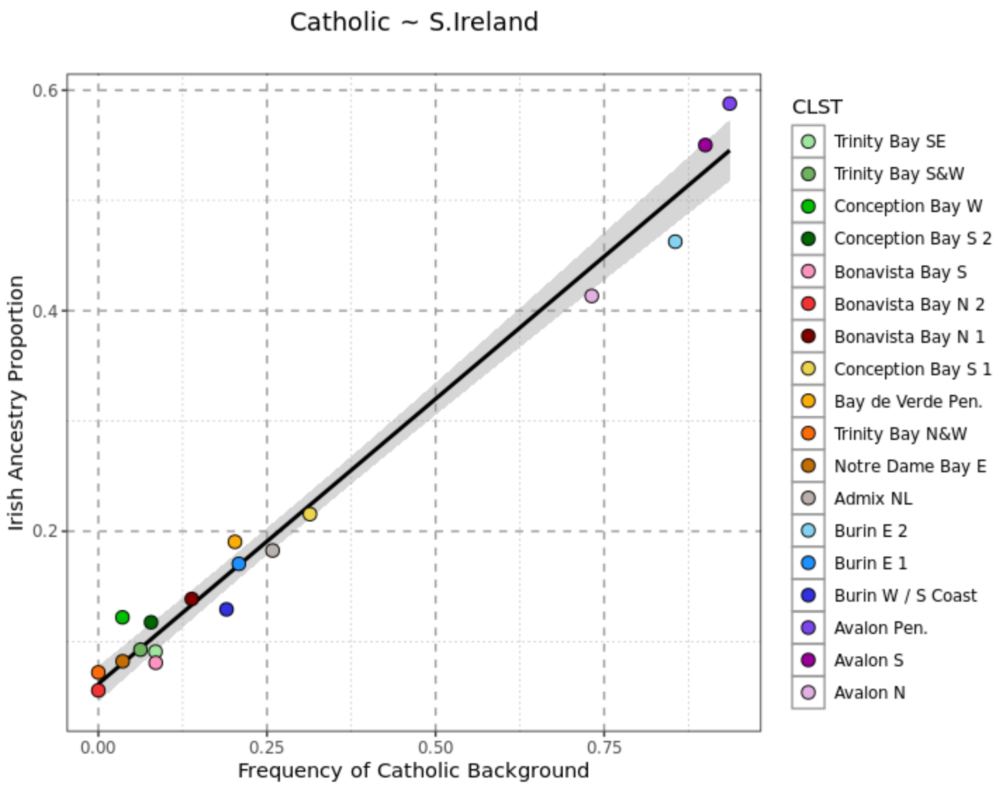

Supp Figure 27 – The per-*fineSTRUCTURE* cluster proportions of Catholic background (x-axis) versus the average estimated Irish ancestry (y-axis). The trend line represents a linear model between the two proportions with 95% confidence shading.

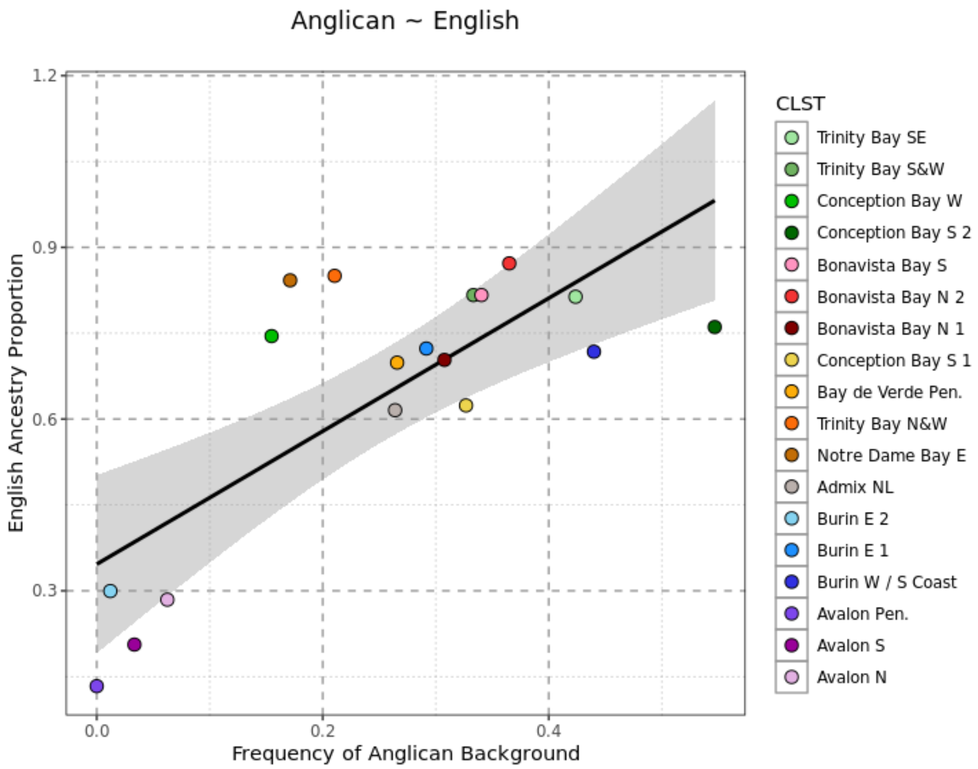

Supp Figure 28 – The per-*fineSTRUCTURE* cluster proportions of Anglican background (x-axis) versus the average estimated English ancestry (y-axis). The trend line represents a linear model between the two proportions with 95% confidence shading.

IBD Copying Profiles Across Ireland, Britain, and NL

In addition to modelling each NL *fineSTRUCTURE* cluster as a mixture of Irish or British haplotypes we generated broad ancestry profiles in an "unsupervised" analysis, investigating overall IBD sharing patterns between NL, Irish, and British clusters. This analysis would detect both within and without sharing between these three populations. Using the methodology set out in Methods, for each *fineSTRUCTURE* or IBD cluster we recorded IBD segments shared between individuals within that cluster and every other *fineSTRUCTURE* or IBD cluster.

We treated each NL, Irish, and British cluster as a target cluster separately, using every other NL, Irish, or British cluster as a source (Supp Figure 26). Visualising these pairwise comparisons of target versus source clusters as a heatmap of average ancestry contributions, relationships between groups of clusters become clear. Each of the three broad population groups (NL, Britain, and Ireland) present evidence of genetic regions of inter-related clustering. The patterns in Ireland and Britain are consistent with previous results[15,19,20]. Within NL, clusters from the Avalon Peninsula region are distinct from the other NL clusters, in agreement with *fineSTRUCTURE* dendrogram ordering. Interestingly, despite high levels of NL-NL IBD sharing patterns, we detected low IBD-sharing in some NL target clusters from British and Irish source clusters. In Avalon for example, this is predominantly from Irish sources, not British. This agrees with the high levels of estimated Irish haplotypes in these NL regions summarised in Figure 3.

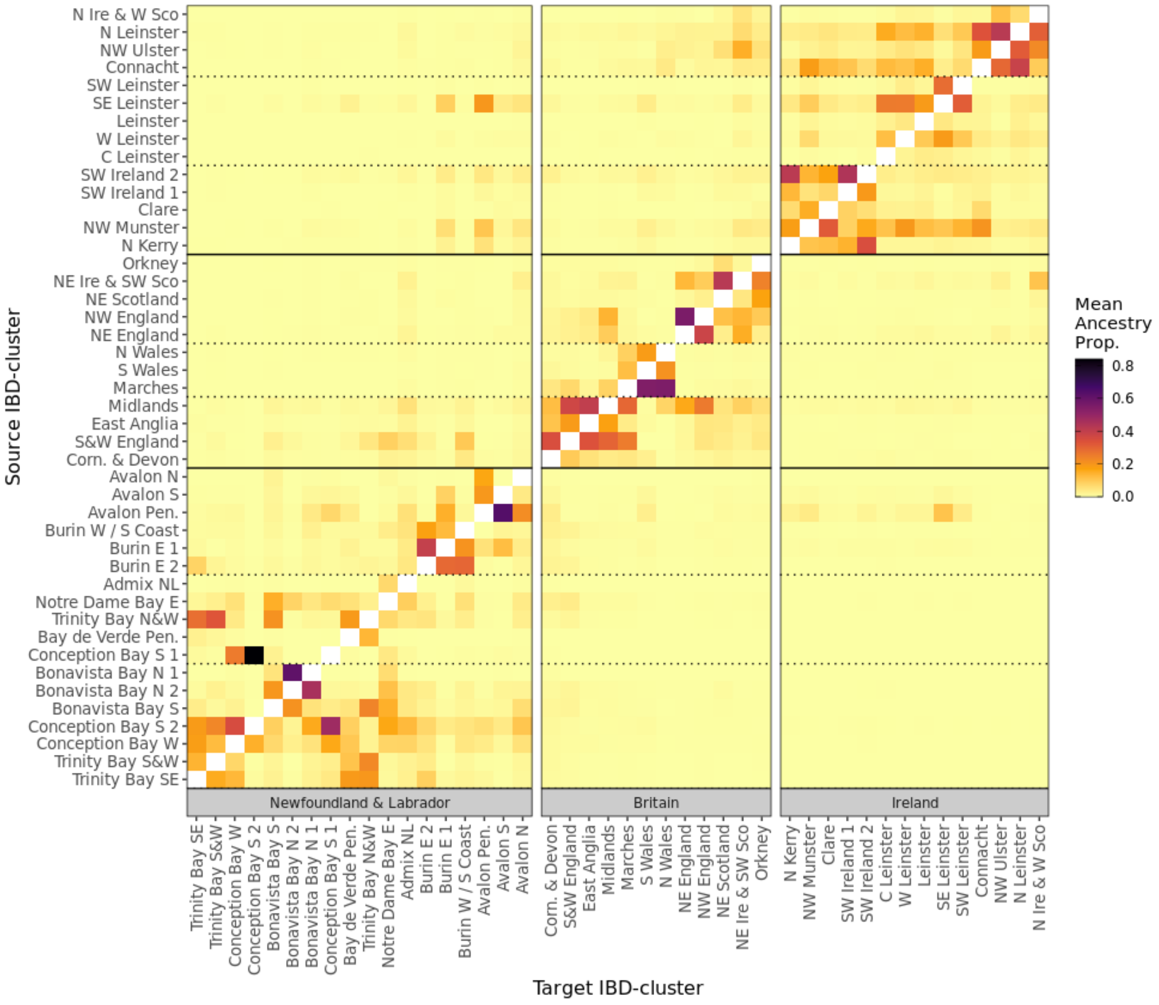

Supp Figure 29 – The mean per-cluster pair "ancestry proportion" matrix calculated using IBD-sharing proportions and the nnls-method[19] between all NL, Irish, and British cluster pairs. Solid horizontal lines indicate groups of overall populations, and dotted horizontal lines indicate broad sub-groups of clusters within those populations.

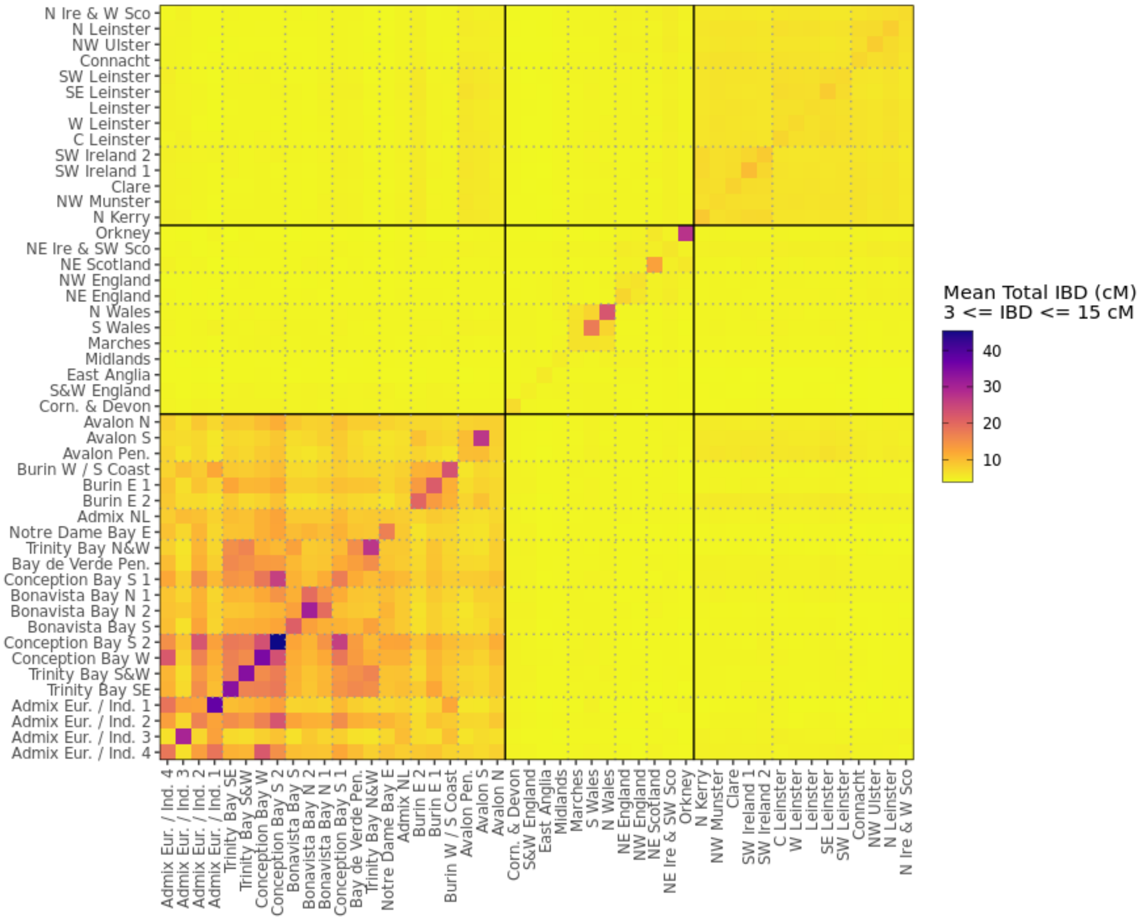

Supp Figure 30 – The average total length of IBD > 3cM and < 15 cM between all NL and Irish and British clusters. Clusters are grouped according to NL, British, or Irish membership. These groups are divided by solid black lines, and groups of clusters within NL, Ireland, or Britain are shown with dotted grey lines.

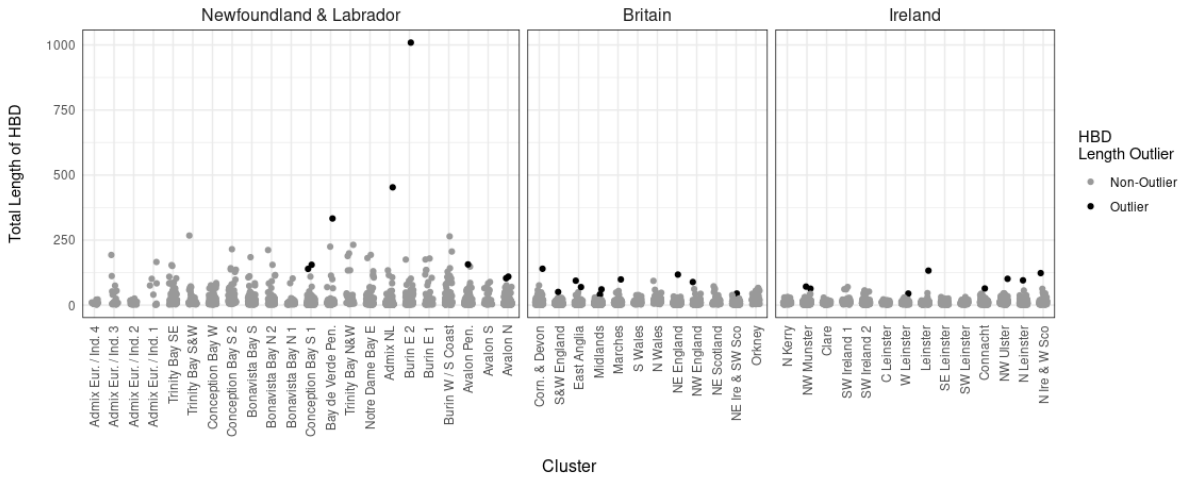

Supp Figure 31 – Total length (cM) of Runs of Homozygosity > 1cM per NL and Ire-Brit individual, grouped according to cluster membership. Each point represents the total length of ROH or "HBD" detected in that individual by *refinedIBD*[21], colour coded according to outlier status. An outlier is an individual whose total ROH is greater than six standard deviations from its cluster's mean.

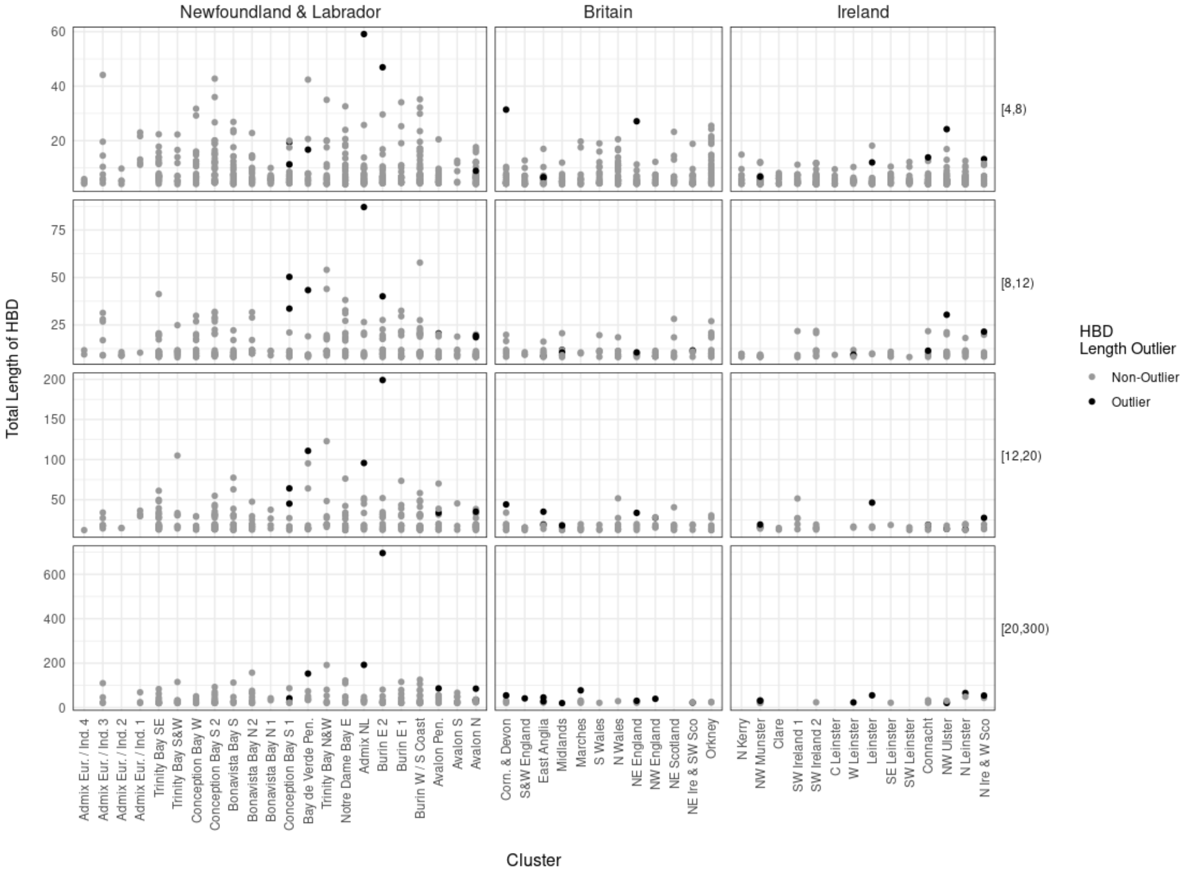

Supp Figure 32 – Total length (cM) of Runs of Homozygosity per NL and Ire-Brit individual, grouped according to cluster membership. Each panel shows the total amount by HBD length bin. Each point represents the total length of ROH or "HBD" detected in that individual by *refinedIBD*[21], colour coded according to outlier status. An outlier is an individual whose total ROH (> 1cM) is greater than six standard deviations from its cluster's mean.

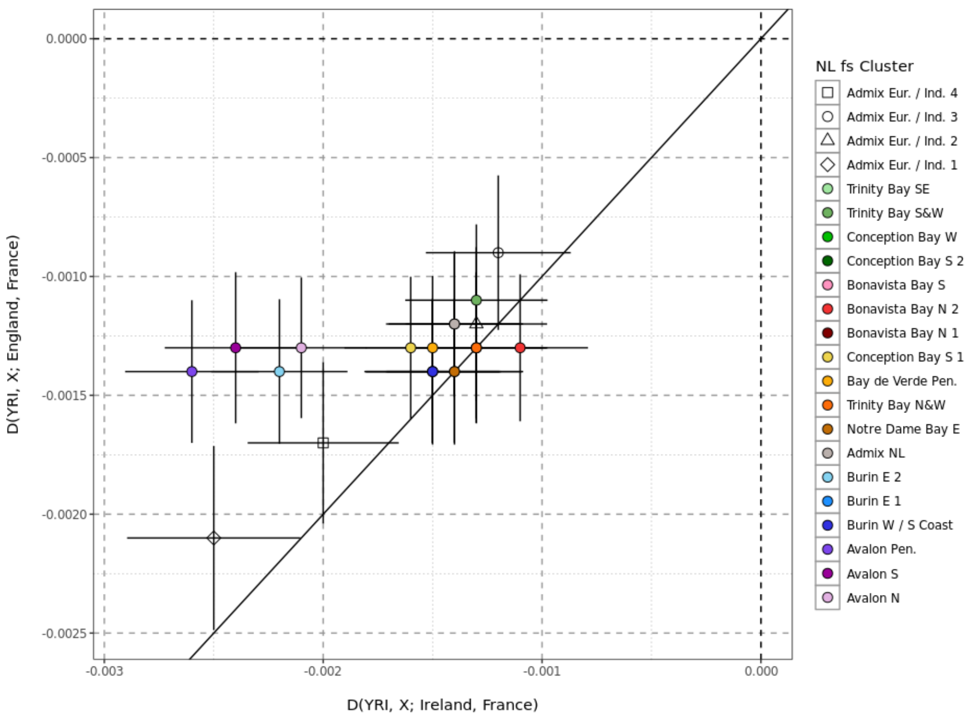

Supp Figure 33 – Patterson's *D* statistic analysis of Irish-English-French ancestry, where negative values on the x and y axes indicate excess allele sharing between NL and Irish or English sources (respectively), and positive values on the x or y axes indicate excess allele sharing between NL and French sources. Error bars shows 1 standard error

Supplemental Note 6

Evidence of Irish-British Admixture in NL from fastGLOBETROTTER

To complement the IBD-based ancestry contribution method reported in our main results we applied the *fastGLOBETROTTER* method to the joint Irish-British and NL dataset. *fastGLOBETROTTER* is an optimised extension of *GLOBETROTTER*[22], providing faster and more accurate inferences of admixture events. We utilised to *fastGLOBETROTTER* to provide evidence of a mixture of Irish and British haplotypes forming the modern European ancestry across NL. We specifically tested the evidence of detectable admixture events between Irish or British source clusters in any NL *fineSTRUCTURE* cluster, and the mixture content of any detected admixture events.

We tested each NL *fineSTRUCTURE* cluster for evidence of a "single-event" admixture signal using the Irish and British IBD-clusters as surrogates for the sources of any detectable event. Using phased haplotype data previously used to detect IBD segments, we generated a *ChromoPainter* co-ancestry "chunklengths" matrix. We "painted" each NL, Irish, or British haplotype as a mixture of every other NL, Irish, or British haplotype using stages 1 and 2 of the *fs* utility[11]. Using the same haplotype data, we generated "painting samples" which are derived samples of the *ChromoPainter* model where each NL individual haplotype was modelled as a mixture of Irish or British haplotypes at each SNP. Leveraging this copying "samples" data, and the total co-ancestry matrix, we performed *fastGLOBETROTTER* analysis, using fastGT mode 1 with 100 bootstraps of the estimated admixture date. We focused on detected "single-event" admixture which had >95% of bootstrap dates replicated within the bounds of accurate *fastGLOBETROTTER* estimation, between 1 and 200 generations ago. We also focussed analysis on events where the admixture model fit well with the underlying data, restricting to event with a maximal R2 goodness-of-fit for the inferred admixture of > 0.5.

As well as estimating admixture in each individual NL *fineSTRUCTURE* cluster, we estimated evidence of admixture using an "all NL" target, which included grouping all NL individuals except for members of an inferred Indigenous-mixed cluster as one NL-meta-cluster. We also did not estimate such admixture in each separate inferred Indigenous-mixed NL cluster as this did not fit the inferred demographic history of these four clusters. We report the co-ancestry curves (Supp Figures 32-50), showing the *fastGLOBETOTTER* model fit to the observed data for each of the tested 19 clusters of NL individuals.

We detect significant evidence of one-date admixture events in 3 of the 18 NL clusters (Supp Figure 31). The average date of these admixture events is 9.5 generations ago, ranging from 9.79 generations in *Avalon N* to 8.59 generations in *Avalon Pen*. Inferred admixture events with the greatest fit to the data (including the results of the "whole NL" analysis) tend to date to 10 generations ago. These tend to predate the historical records of 18th and 19th century settlement, though the *GLOBETROTTER* algorithm's date estimates can be considered as an upper estimate[19], so could be viewed as consistent with the historical record. This analysis also further supports our IBD-based results from *nnls* analysis, where the mixing sources are from the south-east of Ireland (with NW Munster acting as the major surrogate for an Irish source) and the south-west of England (with S&W England acting as the major surrogate for an English source). Together, these results support European ancestry in NL as a mixture of ancestries from the southeast of Ireland and the southwest of England – dating to approximately 10 generations ago.

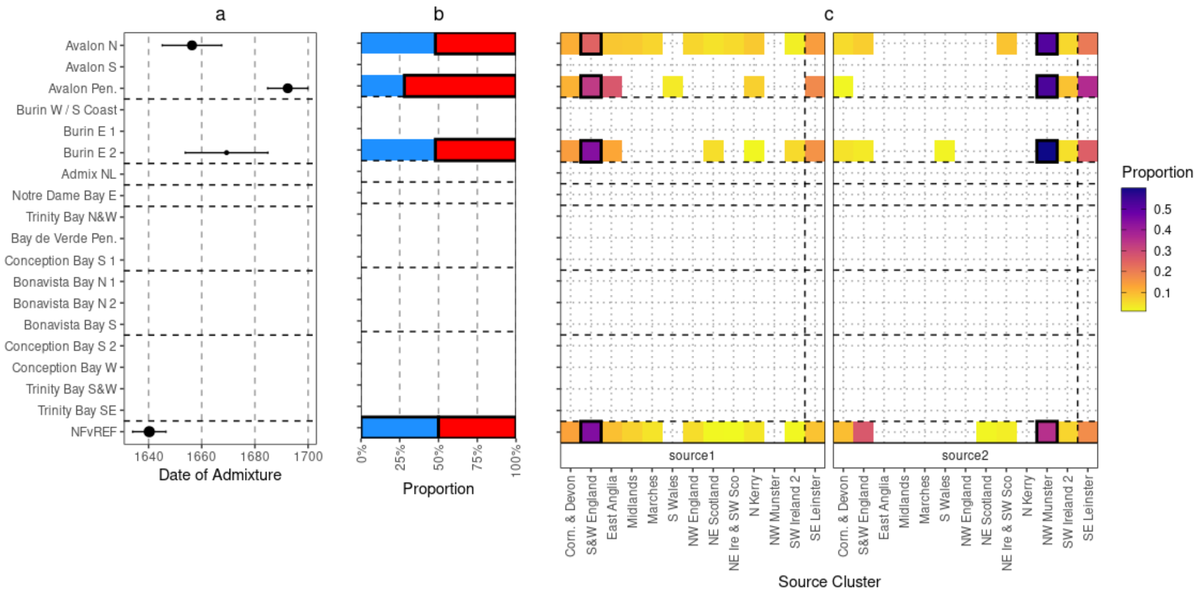

Supp Figure 34 – History of European admixture within Newfoundland and Labrador. (A) Dates of admixture events estimated by *fastGLOBETROTTER* to be a "one-date" event, and where 95% of the estimated dates from bootstrap replicates where >1 or <200 generations ago, and the maximal R2 goodness-of-fit of the inferred admixture event is > 0.5. Error bars show 95% confidence intervals, and the size of point is proportional to the maximal R^2 goodness-of-fit of the inferred admixture event to co-ancestry curves. (B) Inferred proportions of the two mixing sources in each significant estimated one-date admixture event. Blue shows "source1" and red "source2", with a black border showing the majority mixing source. (C) The inferred surrogate sources for source1 and source2, with colour indicating proportion that the surrogate contributes to mixing sources 1 or 2. Black border shows the surrogate which contributes the majority to each mixing source. All panels were plotted using the statistical computing language R[3] and the package ggplot2.

fastGLOBETROTTER Coancestry Curves

Shown below are the co-ancestry curves constructed by *fastGLOBETROTTER* to infer admixture times and the makeup of mixing groups from a list of surrogates. For each NL *fineSTRUCTURE* we show the curves between the surrogates which contributes the majority to each admixing source (highlighted in Supplementary Figure 5.1). Each curve shows the weighted probability that two positions separated by distance X-axis copy from the pair of populations listed above each panel – with red and green lines showing the *fastGLOBETROTTER* inferred fit for the date estimate (red) or mixing source make-up (green).

Supp Figure 35 – NLvREF

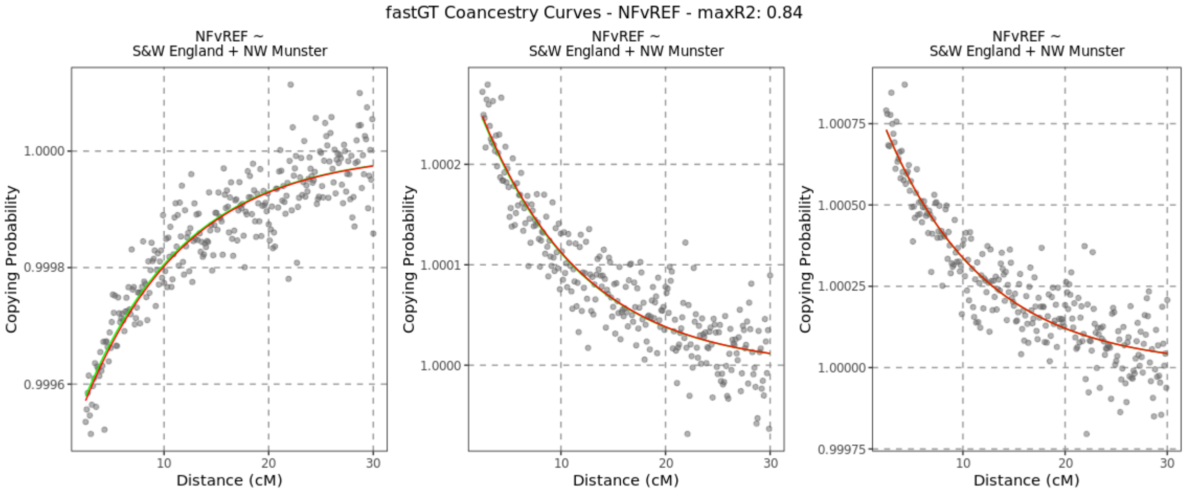

Supp Figure 36 – Avalon N

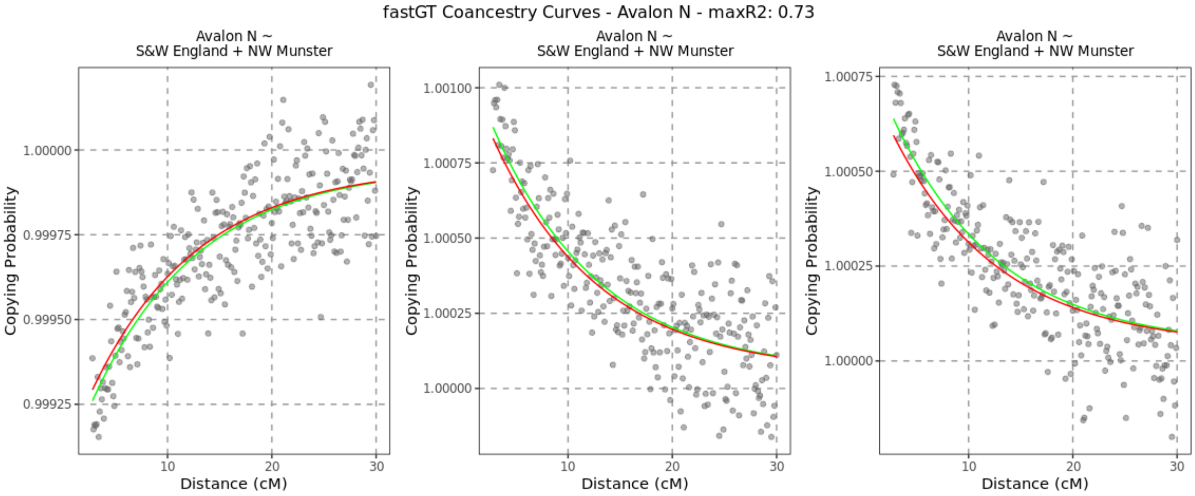

Supp Figure 37 – Avalon S

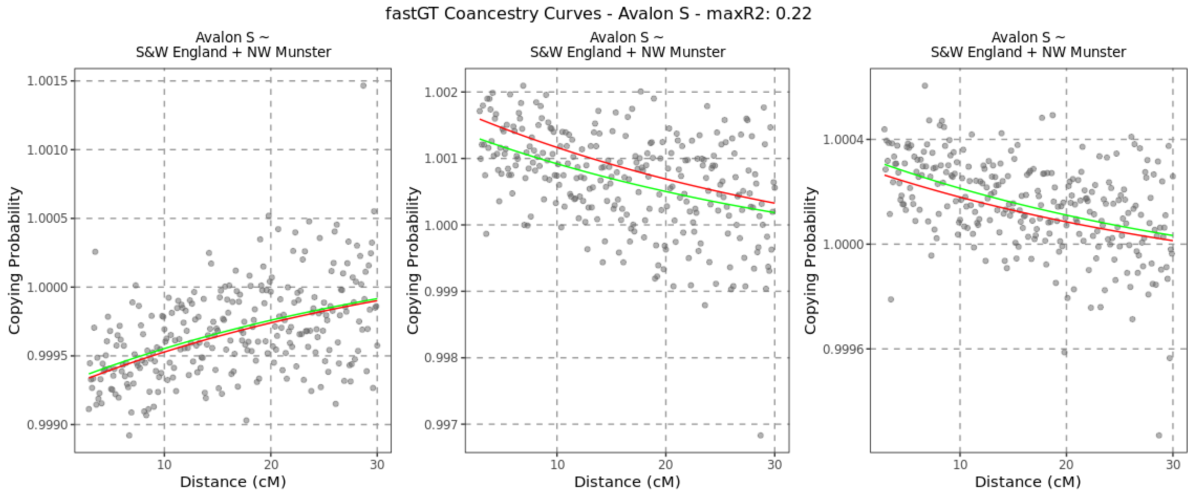

Supp Figure 38 – Avalon Pen.

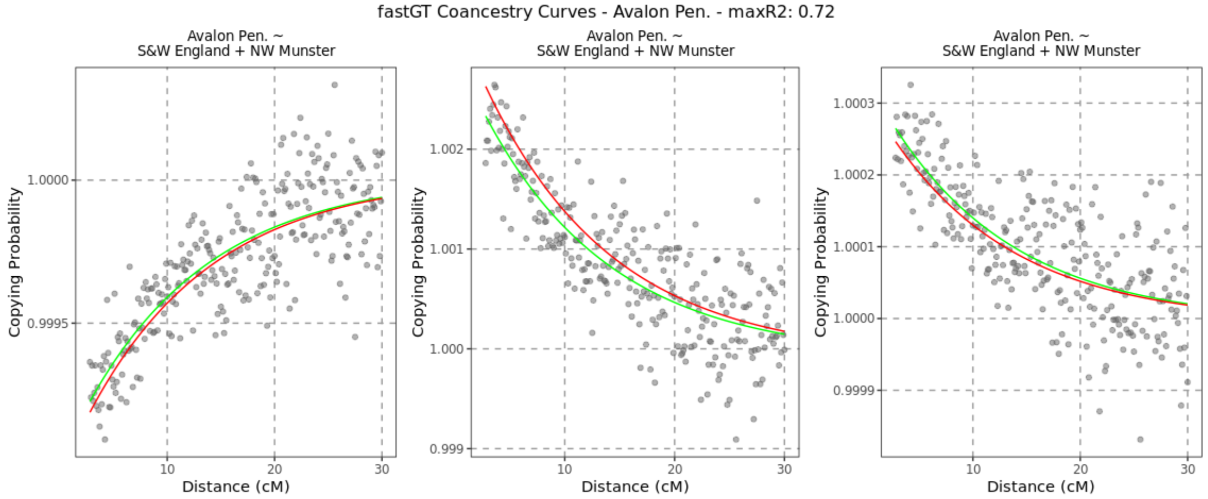

Supp Figure 39 – Burin W/S Coast

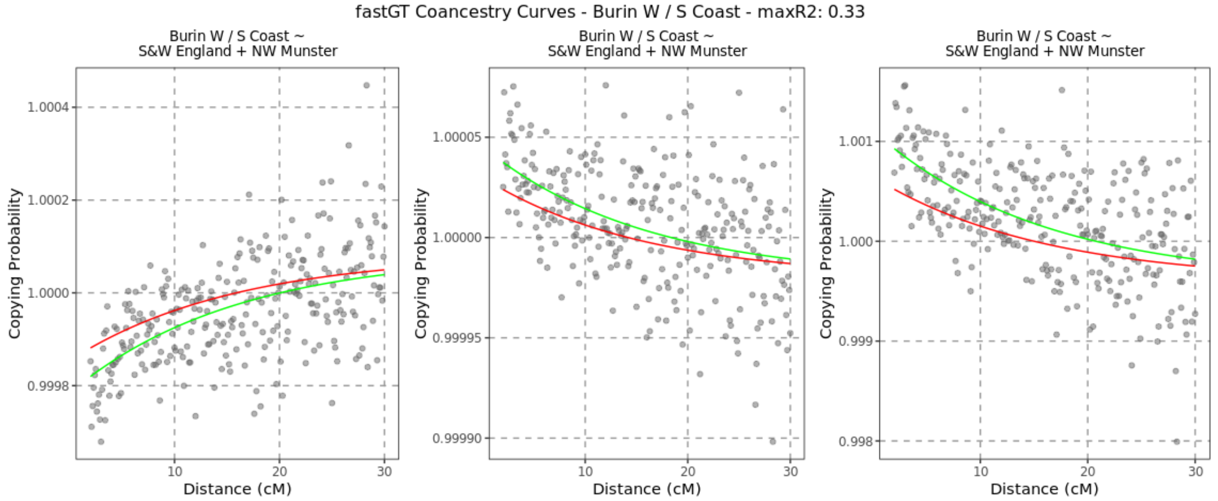

Supp Figure 40 – Burin E 1

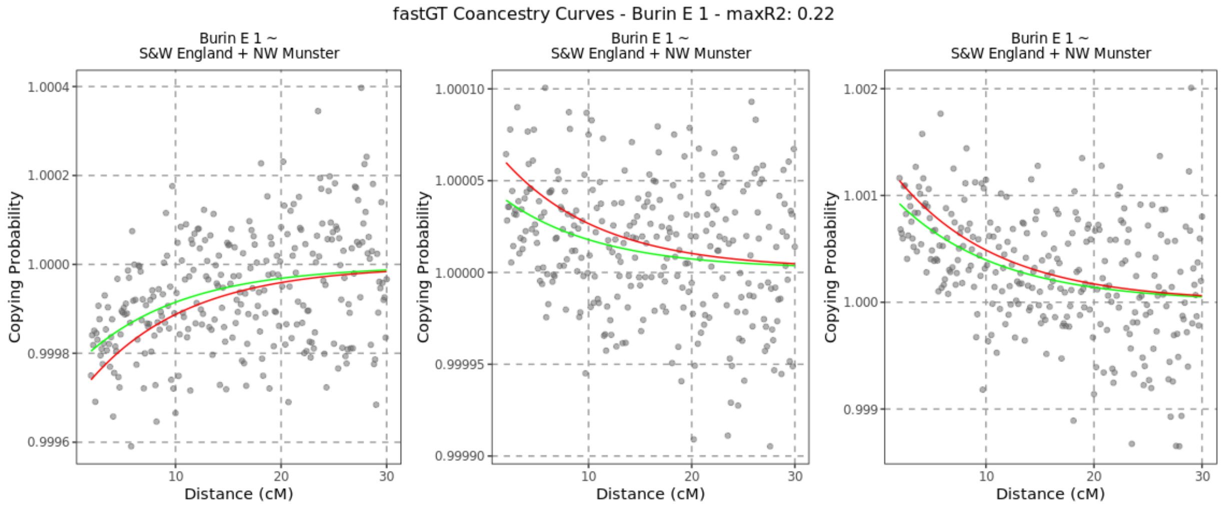

Supp Figure 41 – Burin E 2

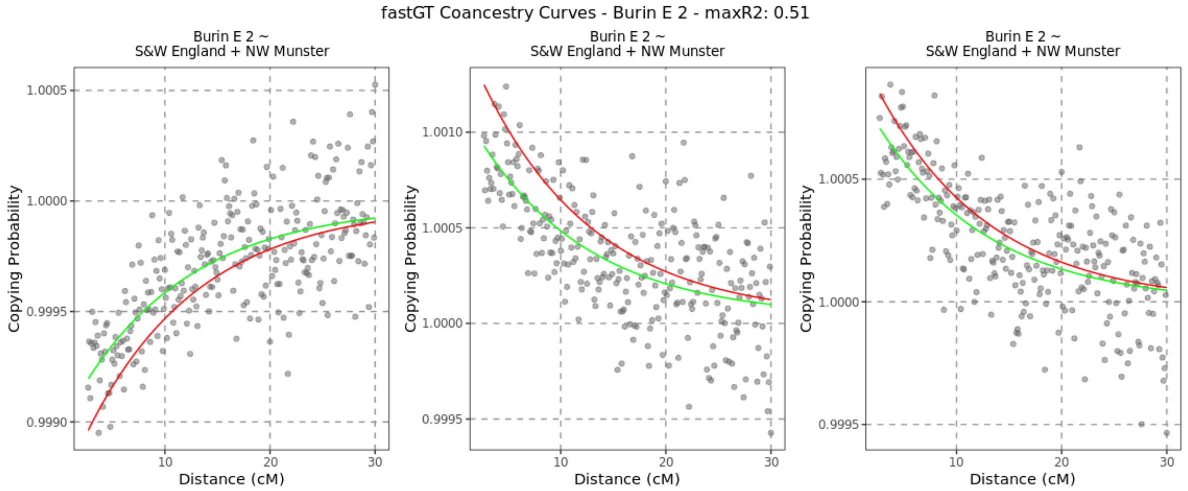

Supp Figure 42 – Admix NL

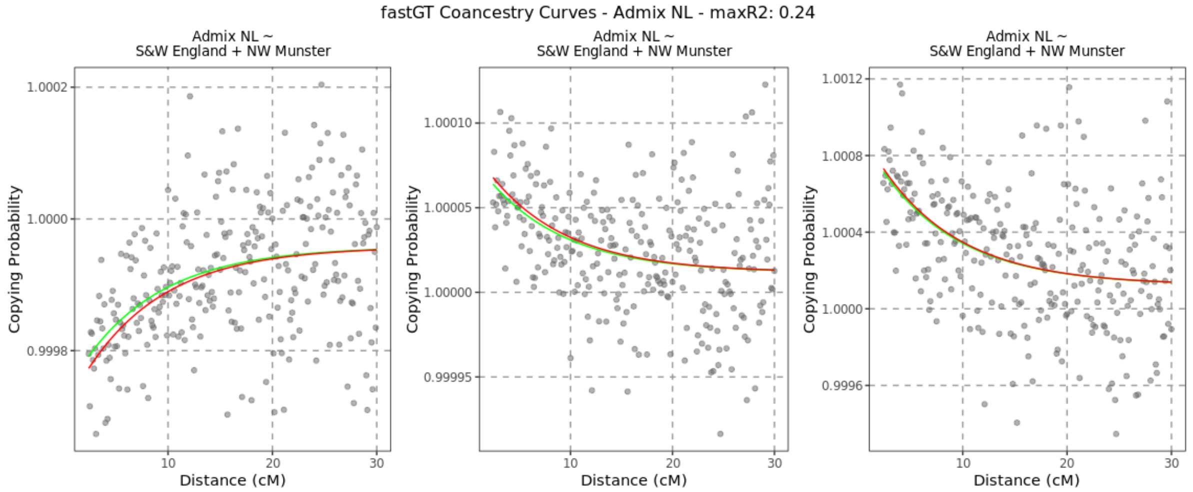

Supp Figure 43 – Notre Dame Bay E

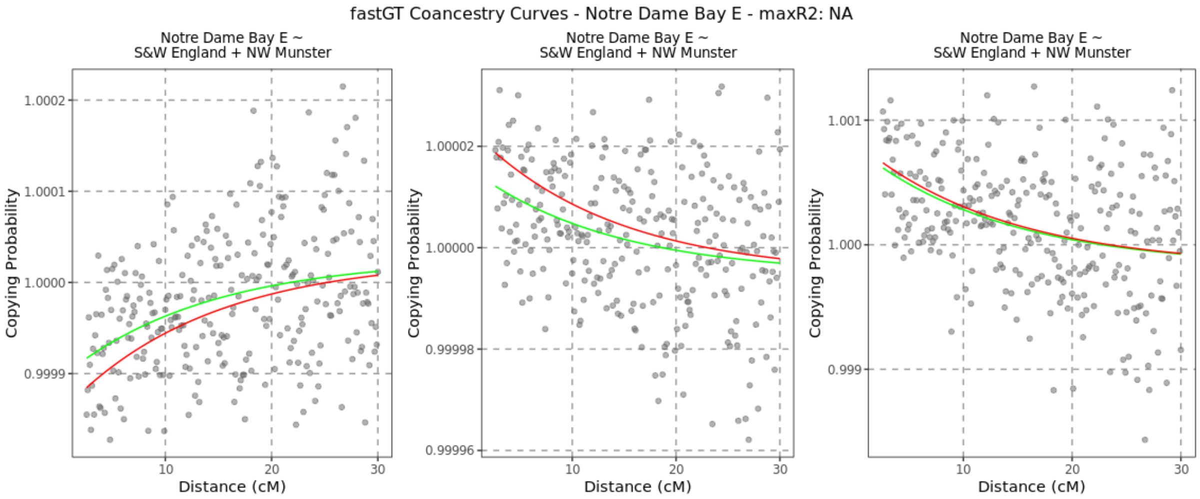

Supp Figure 44 – Trinity Bay N&W

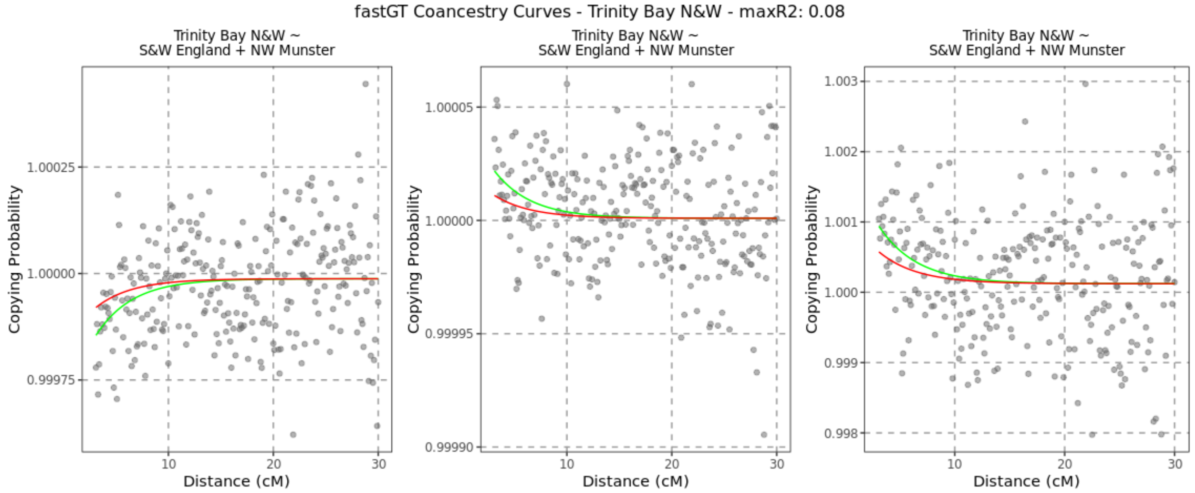

Supp Figure 45 – Bay de Verde Pen.

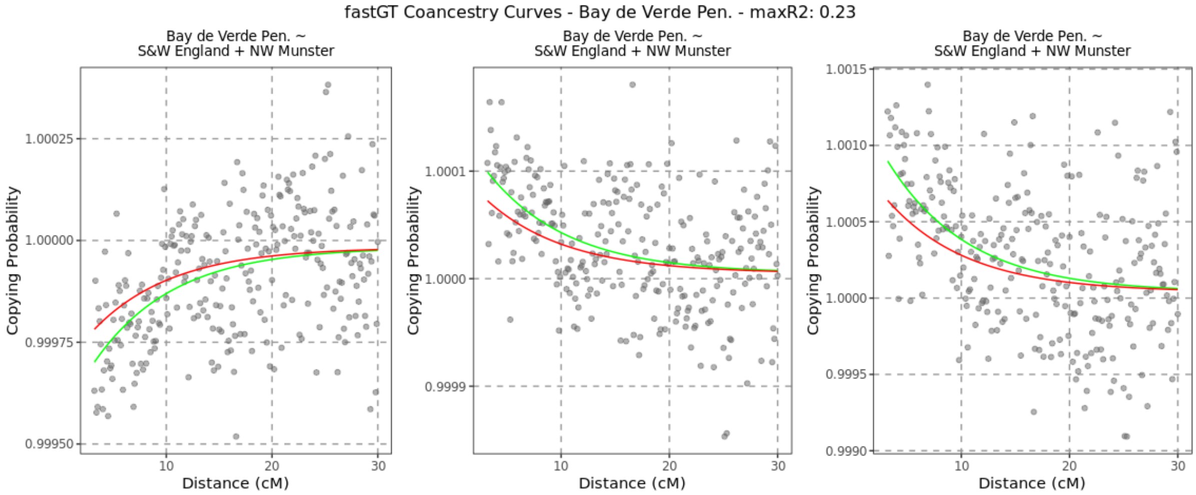

Supp Figure 46 – Concep. Bay S 1

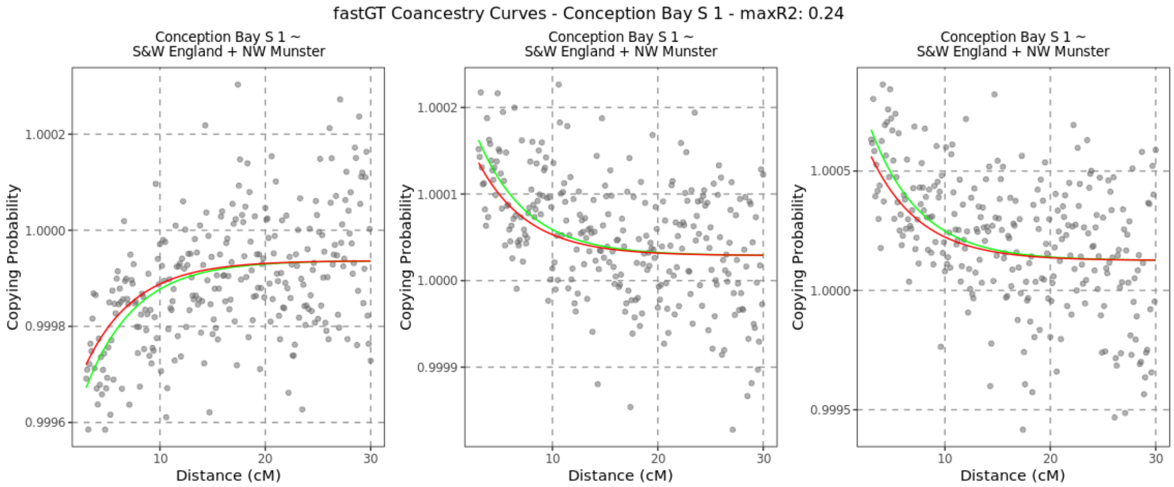

Supp Figure 47 – Bonavista Bay N 1

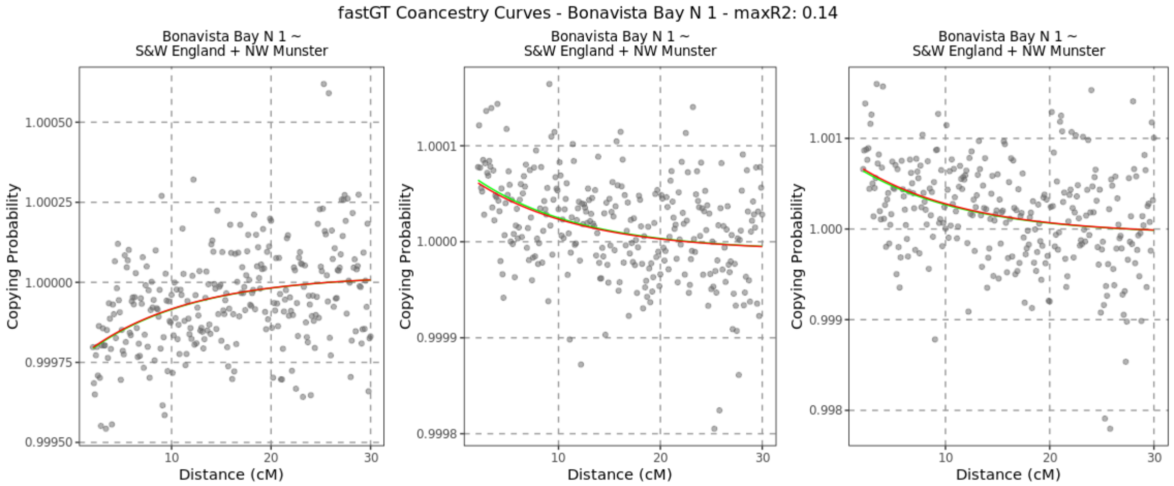

Supp Figure 48 – Bonavista Bay N 2

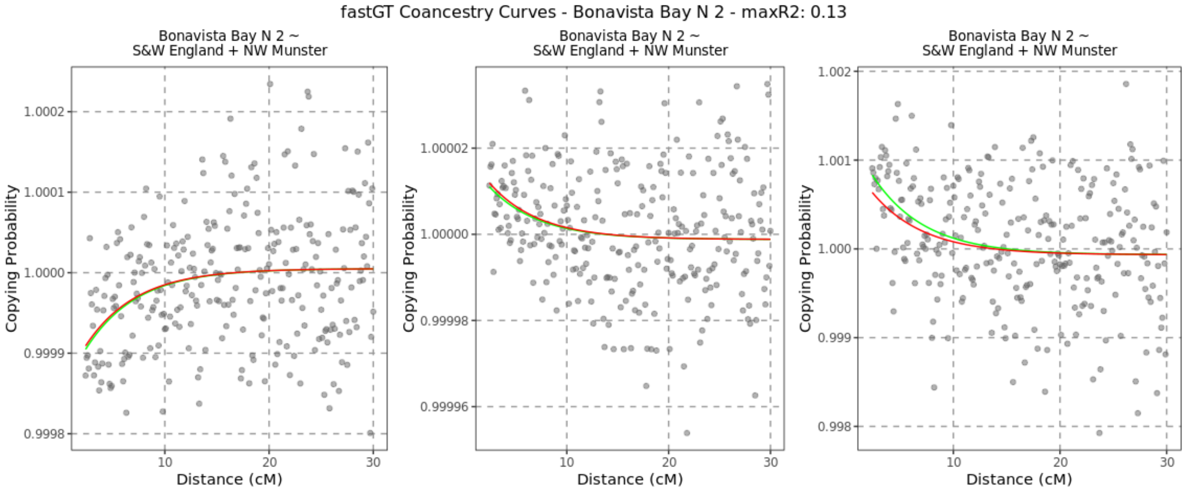

Supp Figure 49 – Bonavista Bay S

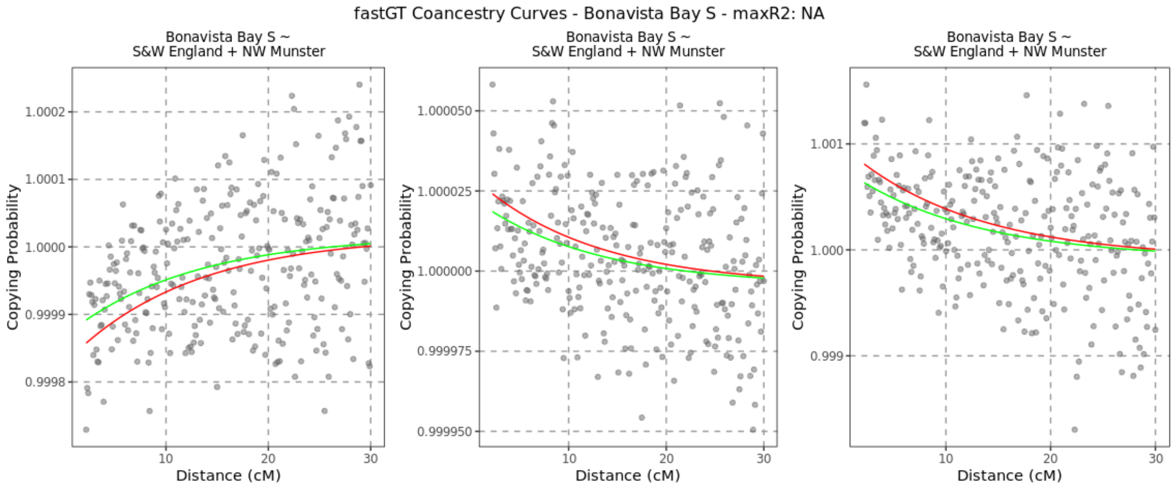

Supp Figure 50 – Conception Bay S 2

Supp Figure 51 – Conception Bay W

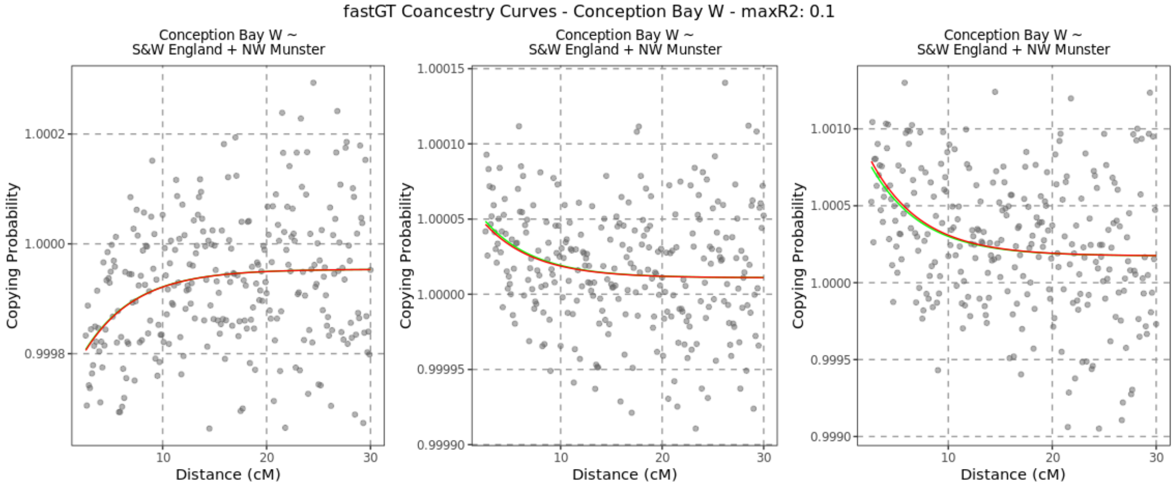

Supp Figure 52 – Trinity Bay SW

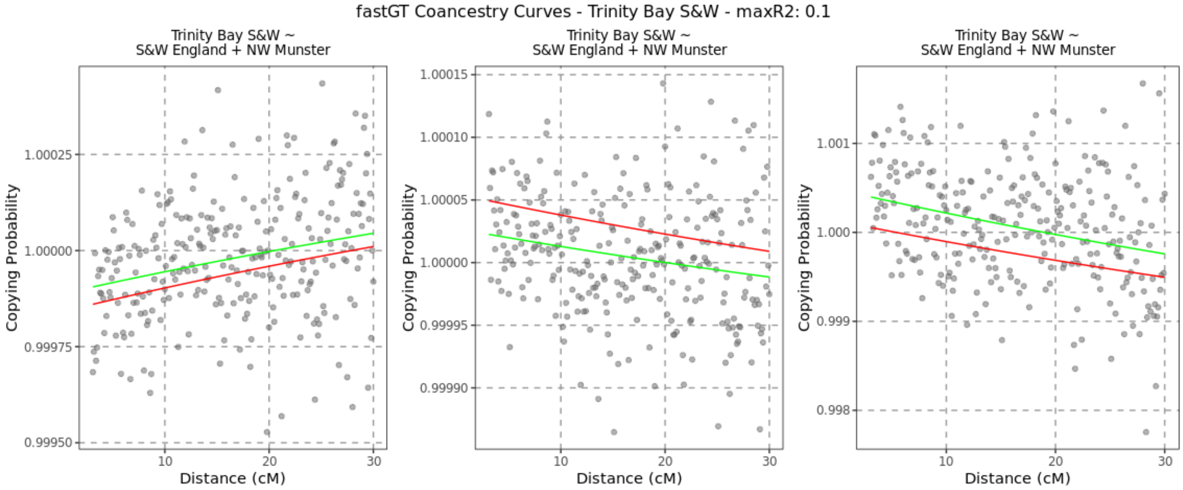

Supp Figure 53 – Trinity Bay SE

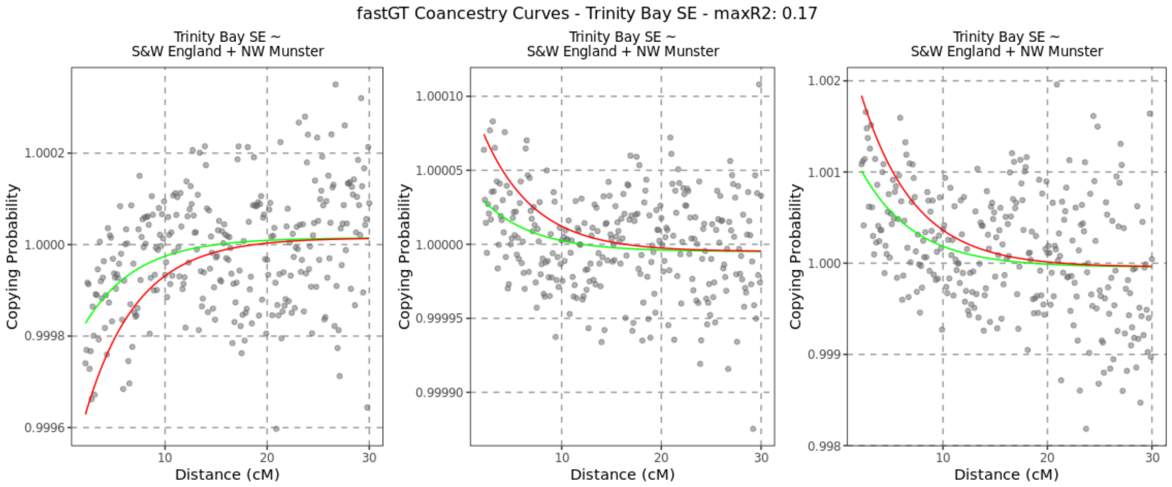
fastGT Coancestry Curves - Trinity Bay SE - maxR2: 0.17
Trinity Bay SE ~
S&W England + NW Munster
Trinity Bay SE ~
S&W England + NW Munster
Trinity Bay SE ~
S&W England + NW Munster
Copying Probability
Distance (cM)
1.0002
1.0000
0.9998
0.9996
1.00010
1.00005
1.00000
0.99995
0.99990
1.002
1.001
1.000
0.999
0.998
10
20
30

Supplementary Note 7

Evaluating IBD Segment Calling

Several of our analyses into the demographic history of NL in comparison to Ireland or Britain necessitated a combined genotype dataset using common SNP markers (n=178,603) which could be considered to be low for effective calling of shorter (1-5 cM) IBD segments[21,23]. This is especially true of algorithms such as IBDNe which assumes input IBD segments to be true[24]. Motivated by this concern we evaluated the results of the IBD segment calling in the NL and Irish-British joint dataset.

First of all, we reran the IBDNe algorithm to estimate recent effective population size using the same parameters as outlined in the Results, but only considering IBD segments of a length ≥7 cM as segment this length have been shown to have an extremely high accuracy[23,25]. The results of analysis are shown in Supp Figure 51, where whilst the population size estimates are consistent with the analysis considering only IBD segments ≥4 cM in length across Ireland and Britain and NL post-putative-bottleneck there are some differences. These are primarily prior to the detected reduction in ancestors at around 10-15 generations in NL clusters > 100 individuals in size where the clusters *Burin W/S Coast* and *Notre Dame Bay E* have historical population sizes more consistent with other NL clusters (i.e., approximately 1 x 10^5) and lower confidence intervals – which would be concordant with higher confidence IBD segment input data. We observe no substantial difference in our Irish or British estimates. Our population size estimates using high quality IBD segment here support our main findings, in particular the signal of a substantial reduction on ancestors in NL clusters, which all show evidence of varying sized bottlenecks.

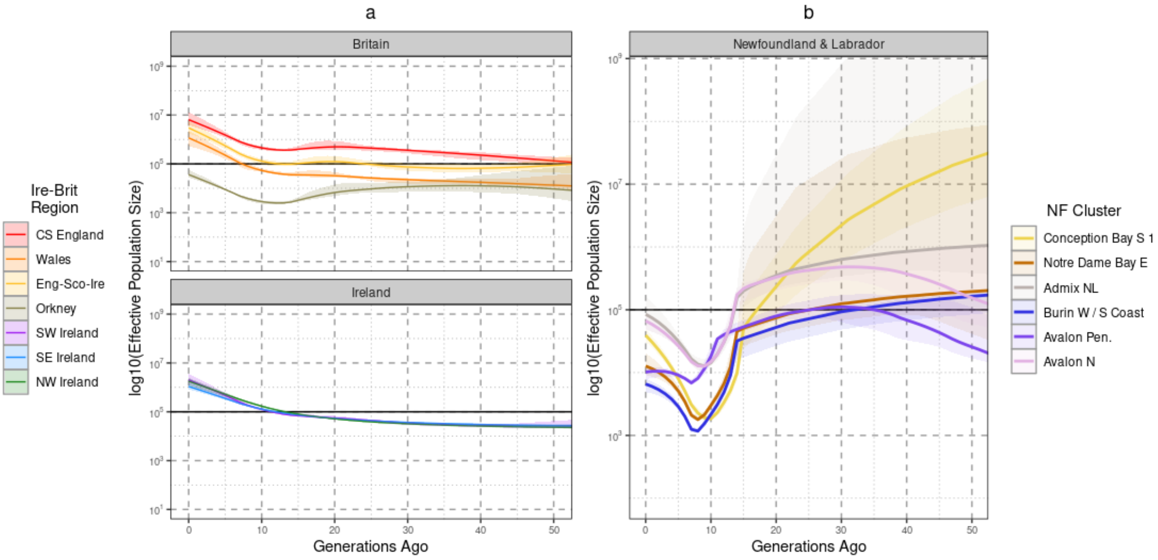

Supp Figure 54 – The recent effective population size estimates of NL genetic clusters > 100 individuals in membership size and Irish and British regions using IBDNe[24] and only IBD segments ≥7 cM in length. Shading shows the 95% confidence intervals.

In addition to re-estimating historical effective population size using high-confidence IBD segments we compared IBD-segment sharing estimates within and between NL *fineSTRUCTURE* clusters using

two difference SNP sets. The first set were the common markers between the NL, Irish, and British references (n=178,603), and the second were the markers common between the NL genotypes only (n=685,221). We detected the agreement between the average individual-pair normalised total length of IBD within an NL cluster, and the average individual-pair normalised total length of IBD between pairs of NL clusters – using same methods as Figure 4C and Supp Figure 26. We calculated this agreement using three bins of IBD segment length; [1,3), [3,5), and [5-7)[23] – as well as using all segments > 1 cM in length. (Supp Figures 52-54). We find good agreement between the estimates of total length of IBD between cluster pairs over the two SNP-sets (r^2 values 0.921 – 0.995) and are all significantly correlated as tested by the *cor.test()* function in R[3]. We observe a consistent under-estimate of total IBD in the reduced SNP-set (Supp Figure 52), which is largely driven by the [1,3) length bin (Supp Figure 53) – which would be consistent with a lower density of markers making It more difficult to call shorter IBD segments. We find good general agreement of absolute values in the [3,5) and [5,7) bins (Supp Figures 54-55), which supports our approach in calculating effective population size, as well as out *nnls* approach in estimate ancestry proportions.

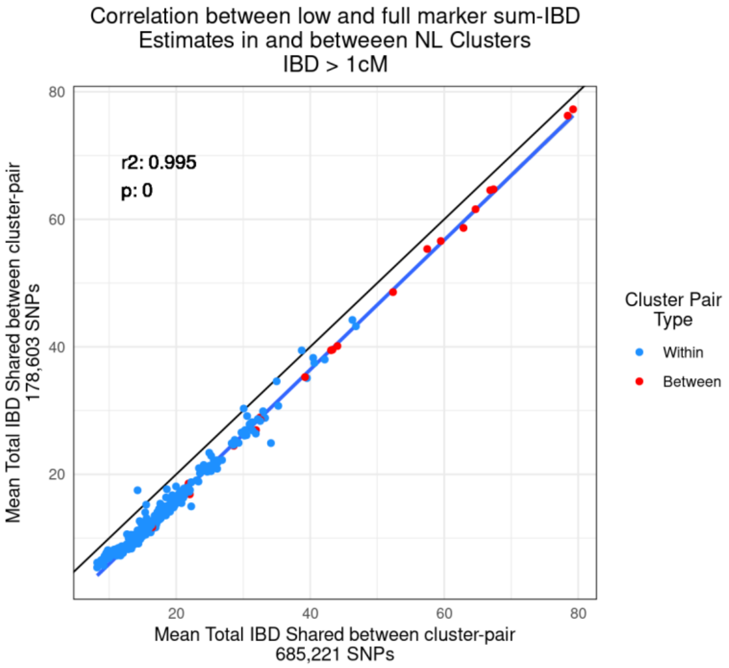

Supp Figure 55 – Agreement between within- and between- NL *fineSTRUCTURE*[11] cluster IBD sharing using two different SNP-sets; one of 685,221 SNPs (x-axis) and another set (y-axis) of common 178,603 SNPs between NL genotypes, and Irish and British reference genotypes. Each point is an estimate for a specific NL cluster pair, with between and within estimates shown in red or blue, respectively. The solid line shows x=y (i.e., perfect agreement). The blue line with shading shows the smoothed fitted line between these points using the lm method and formula of y~x. Show in upper left are the r^2 and p-value from the R[3] *cor.test()* statistical test of correlation between the two estimates of total IBD shared (cM).

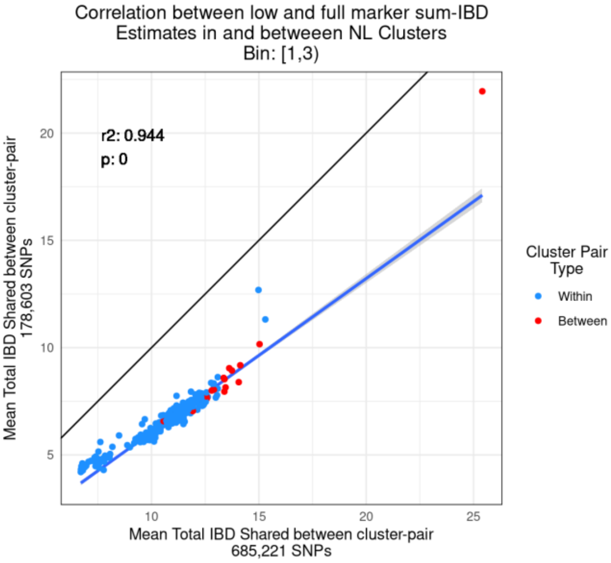

Supp Figure 56 - Agreement between within- and between- NL *fineSTRUCTURE*[11] cluster IBD sharing using two different SNP-sets; one of 685,221 SNPs (x-axis) and another set (y-axis) of common 178,603 SNPs between NL genotypes, and Irish and British reference genotypes. We show the distribution using IBD segments of the specific length bin [1,3). Each point is an estimate for a specific NL cluster pair, with between and within estimates shown in red or blue, respectively. The solid line shows x=y (i.e., perfect agreement). The blue line with shading shows the smoothed fitted line between these points using the lm method and formula of y~x. Show in upper left are the r^2 and p-value from the R[3] *cor.test()* statistical test of correlation between the two estimates of total IBD shared (cM).

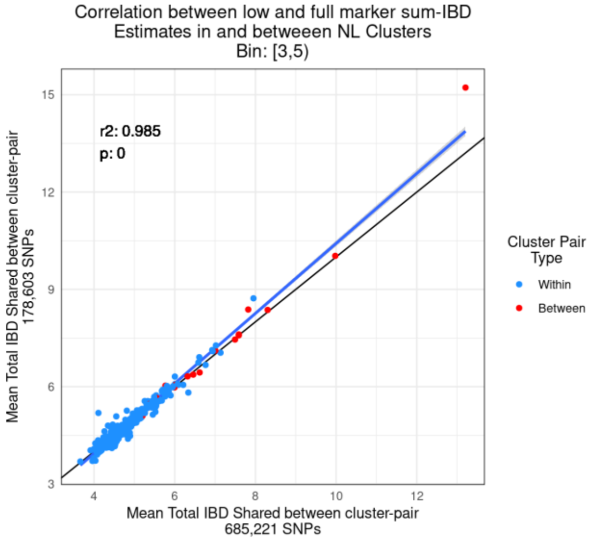

Supp Figure 57 - Agreement between within- and between- NL *fineSTRUCTURE*[11] cluster IBD sharing using two different SNP-sets; one of 685,221 SNPs (x-axis) and another set (y-axis) of common 178,603 SNPs between NL genotypes, and Irish and British reference genotypes. We show the distribution using IBD segments of the specific length bin [3,5). Each point is an estimate for a specific NL cluster pair, with between and within estimates shown in red or blue, respectively. The solid line shows x=y (i.e., perfect agreement). The blue line with shading shows the smoothed fitted line between these

points using the lm method and formula of y~x. Show in upper left are the r^2 and p-value from the R[3] *cor.test()* statistical test of correlation between the two estimates of total IBD shared (cM).

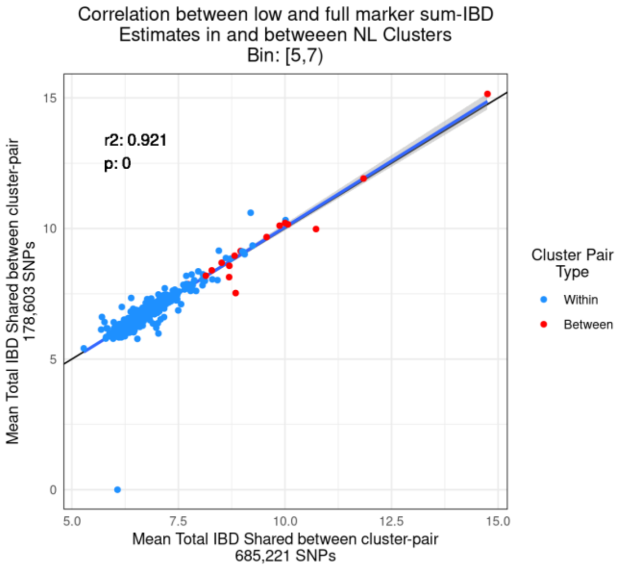

Supp Figure 58 - Agreement between within- and between- NL *fineSTRUCTURE*[11] cluster IBD sharing using two different SNP-sets; one of 685,221 SNPs (x-axis) and another set (y-axis) of common 178,603 SNPs between NL genotypes, and Irish and British reference genotypes. We show the distribution using IBD segments of the specific length bin [5,7). Each point is an estimate for a specific NL cluster pair, with between and within estimates shown in red or blue, respectively. The solid line shows x=y (i.e., perfect agreement). The blue line with shading shows the smoothed fitted line between these points using the lm method and formula of y~x. Show in upper left are the r^2 and p-value from the R[3] *cor.test()* statistical test of correlation between the two estimates of total IBD shared (cM).

Finally, we investigated the distribution of IBD segments and ROH calculated in the NL-Irish-British dataset (n=178,603). We would expect these distributions to be roughly approximate in NL outside of consanguinity[26], which we do not detect evidence of in NL (Figure 4D). For the three bins [1,3), [3,5), and [5,7) we calculated the distribution of IBD and HBD ("homozygous by descent", i.e., Runs of Homozygosity detected by *refinedIBD*) over different segment lengths.

We estimated the confidence of these distributions through bootstrapping where each bootstrap replicate, we randomly sample 22 autosomes with replacement – calculating the overall per-individual IBD/HBD sharing totals using this random sample. We estimated the distribution of total IBD and HBD between pairs of NL individuals, in bins of 5 cM, calculating the 95% confidence intervals from 100 bootstrap replicates.

We show the results of these distributions in Supp Figures 56-58. We find over the three IBD segment length categories there is good overall agreement with the distribution of IBD and HBD with no substantial deviations that would be suggestive of consanguinity or technical issues arising from false-positive, limited power to detect, and uncertainty of length estimates. Whilst the SNP-set from the NL-Irish-British analysis (n=178,603) is undoubtedly smaller than a SNP-set from one genotype array source (≥ 600,000 SNPs), we find little evidence of substantial deviations of our overall IBD-segment sharing estimates as a result of this. Further any differences are driven by segments between 1 and 3 cM, which are appreciated to be detected with lower accuracy[21,25,27,28].

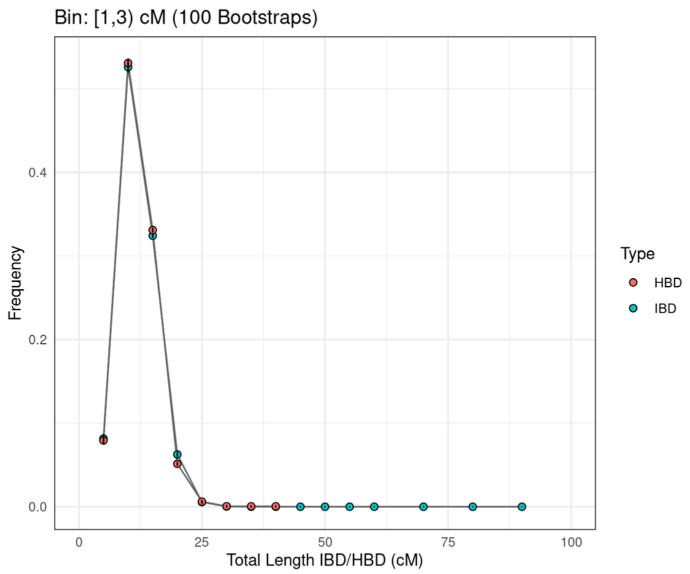

Supp Figure 59 – The distribution of total IBD (blue) or HBD (red) between pairs of NL individuals. Error bars shown are 95% confidence intervals and are estimated from bootstrapping total IBD/HBD length (cM) over 100 replicates of IBD/HBD segments over 22 randomly sampled autosomes with replacement. We show the distribution of total IBD and HBD over the IBD/HBD segment length bin [1,3).

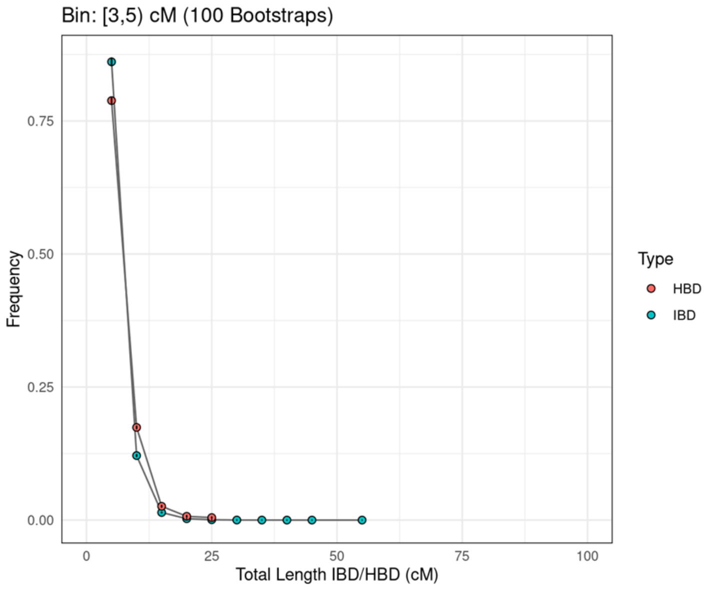

Supp Figure 60 – The distribution of total IBD (blue) or HBD (red) between pairs of NL individuals. Error bars shown are 95% confidence intervals and are estimated from bootstrapping total IBD/HBD length (cM) over 100 replicates of IBD/HBD segments over 22 randomly sampled autosomes with replacement. We show the distribution of total IBD and HBD over the IBD/HBD segment length bin [3,5).

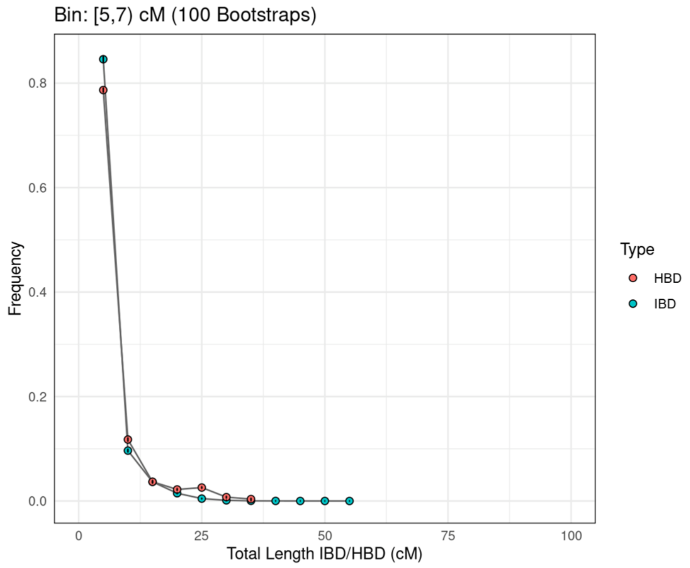

Supp Figure 61 – The distribution of total IBD (blue) or HBD (red) between pairs of NL individuals. Error bars shown are 95% confidence intervals and are estimated from bootstrapping total IBD/HBD length (cM) over 100 replicates of IBD/HBD segments over 22 randomly sampled autosomes with replacement. We show the distribution of total IBD and HBD over the IBD/HBD segment length bin [5,7).

Supplemental Note 8

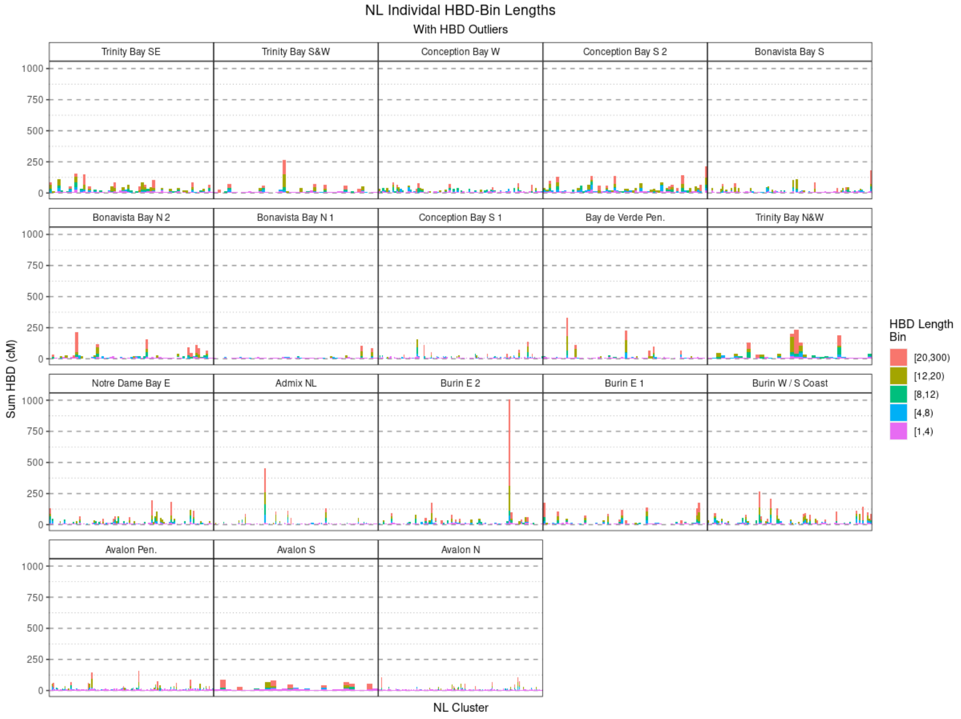

Supp Figure 62 - The per-individual sum of HBD (Homozygous-by-Descent) segment length over five length categories or bins in each of the European-ancestry NL *fineSTRUCTURE* clusters. We have included outlier individuals with excess homozygosity.

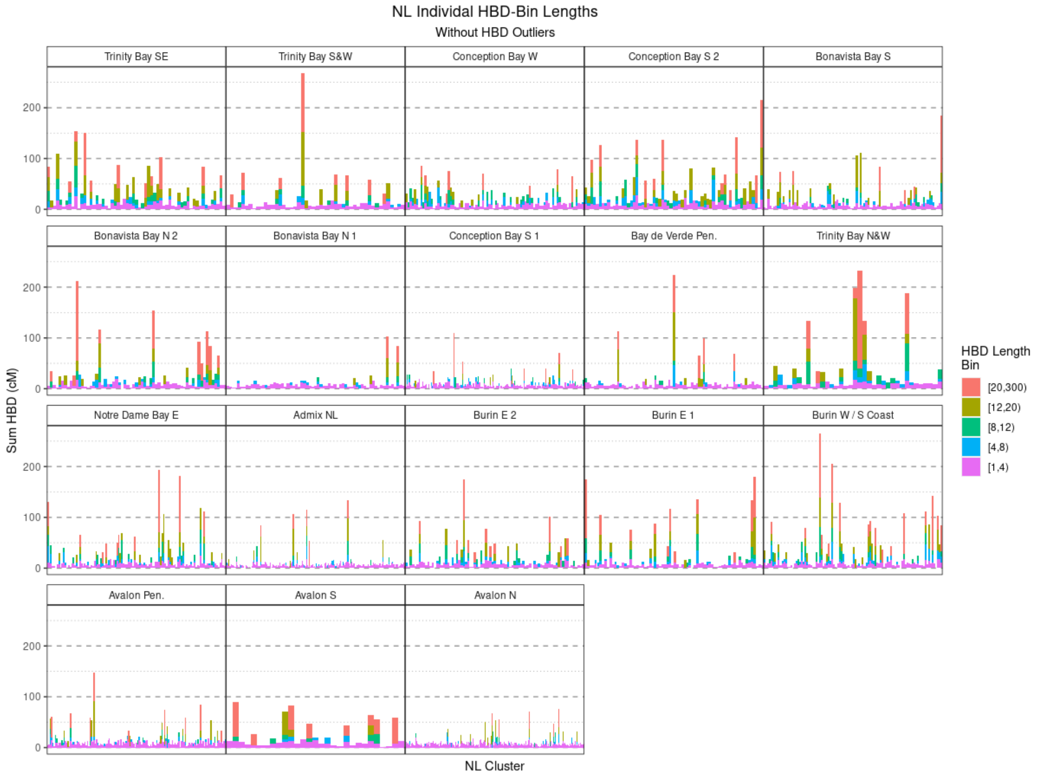

Supp Figure 63 - The per-individual sum of HBD (Homozygous-by-Descent) segment length over five length categories or bins in each of the European-ancestry NL *fineSTRUCTURE* clusters. We have omitted outlier individuals with excess homozygosity to aid visualisation and legibility.

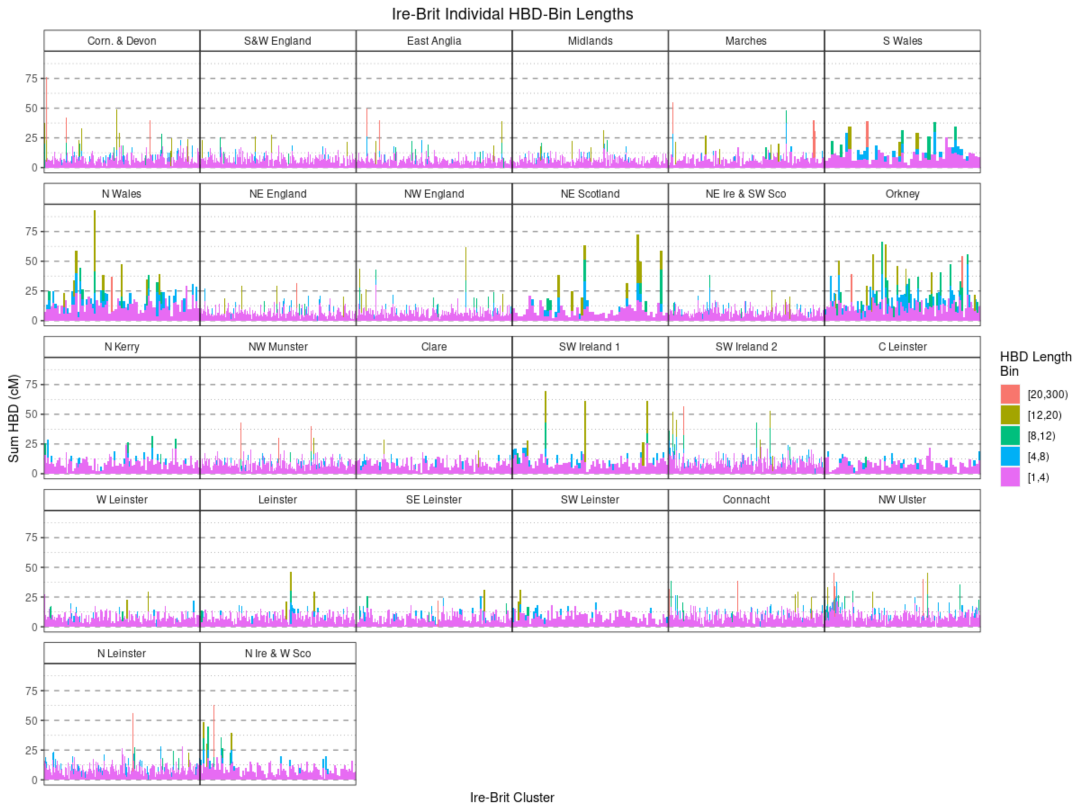

Supp Figure 64 - The per-individual sum of HBD (Homozygous-by-Descent) segment length over five length categories or bins in each of the Irish or British reference clusters for comparison to NL. We note the different Y-axis scale limits specific to the Irish-British results.

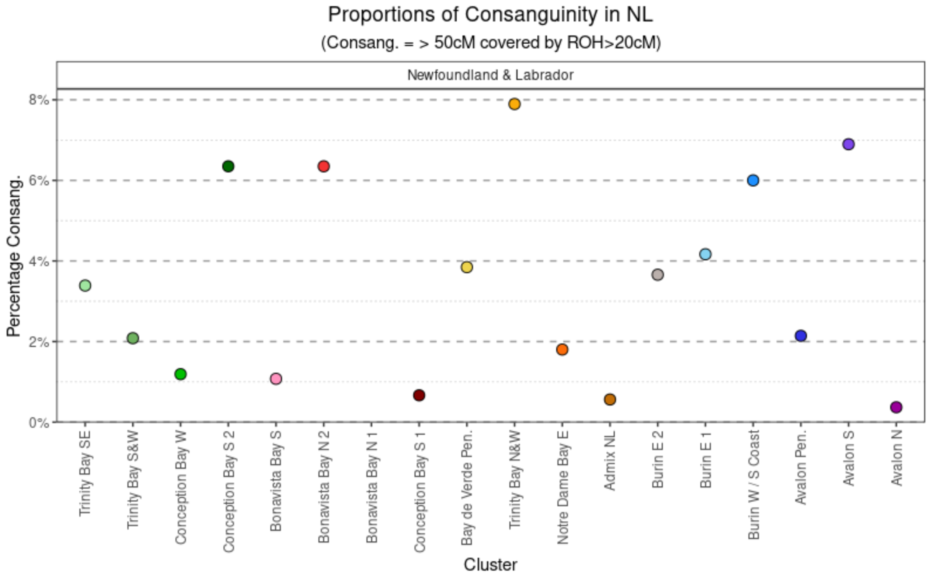

Supp Figure 65 - The per-European-ancestry NL *fineSTRUCTURE* cluster proportions of individuals classed as resulting from consanguinity. We define this as having more than 50 cM of their genome covered by Runs of Homozygosity which are individually > 20 cM in length.

Supplementary References

1. Genomes Project, C. *et al.* An integrated map of genetic variation from 1,092 human genomes. *Nature* **491**, 56-65 (2012).
2. Li, J.Z. *et al.* Worldwide human relationships inferred from genome-wide patterns of variation. *Science* **319**, 1100-4 (2008).
3. Team., R.C. R: A language and environment for statistical computing. *R Foundation for Statistical Computing.* (2017).
4. Csardi G & Nepusz, T. The igraph software package for complex network research. *Complex Systems* **1695**(2006).
5. Wickham, H. ggplot2: Elegant Graphics for Data Analysis. *Springer-Verlag New York.* (2016.).
6. Zhai, G. *et al.* Genetic structure of the Newfoundland and Labrador population: founder effects modulate variability. *Eur J Hum Genet* **24**, 1063-70 (2016).
7. Purcell, S. *et al.* PLINK: a tool set for whole-genome association and population-based linkage analyses. *Am J Hum Genet* **81**, 559-75 (2007).
8. Chang, C.C. *et al.* Second-generation PLINK: rising to the challenge of larger and richer datasets. *Gigascience* **4**, 7 (2015).
9. Alexander, D.H., Novembre, J. & Lange, K. Fast model-based estimation of ancestry in unrelated individuals. *Genome Res* **19**, 1655-64 (2009).
10. Genomes Project, C. *et al.* A global reference for human genetic variation. *Nature* **526**, 68-74 (2015).
11. Lawson, D.J., Hellenthal, G., Myers, S. & Falush, D. Inference of population structure using dense haplotype data. *PLoS Genet* **8**, e1002453 (2012).
12. McInnes, L. & Healy, J. UMAP: Uniform Manifold Approximation and Projection for Dimension Reduction. *ArXiv e-prints* (2018).
13. van der Maaten, L.J.P. & Hinton, G.E. Visualizing high-dimensional data using t-SNE. *J. Mach. Learn. Res* **9**, 2579–605 (2008).
14. Petkova, D., Novembre, J. & Stephens, M. Visualizing spatial population structure with estimated effective migration surfaces. *Nat Genet* **48**, 94-100 (2016).
15. Gilbert, E. *et al.* The Irish DNA Atlas: Revealing Fine-Scale Population Structure and History within Ireland. *Sci Rep* **7**, 17199 (2017).
16. Gilbert, E. *et al.* The genetic landscape of Scotland and the Isles. *Proc Natl Acad Sci U S A* **116**, 19064-19070 (2019).
17. Matsunami, M. *et al.* Fine-Scale Genetic Structure and Demographic History in the Miyako Islands of the Ryukyu Archipelago. *Mol Biol Evol* **38**, 2045-2056 (2021).
18. Newfoundland. Executive Council. Central Statistical Services., Newfoundland. Department of Public Works and Services. Division of Printing Services., Newfoundland. Department of Finance. Economics and Statistics Division., Newfoundland. Department of Finance. Fiscal Policy Division. & Newfoundland Statistics Agency. Historical statistics of Newfoundland and Labrador. volumes (Published under the authority of president of the Executive Council : Printed by Division of Printing Services, St. John's, Nfld., 1994).
19. Leslie, S. *et al.* The fine-scale genetic structure of the British population. *Nature* **519**, 309-314 (2015).
20. Byrne, R.P. *et al.* Insular Celtic population structure and genomic footprints of migration. *PLoS Genet* **14**, e1007152 (2018).
21. Browning, B.L. & Browning, S.R. Improving the accuracy and efficiency of identity-by-descent detection in population data. *Genetics* **194**, 459-71 (2013).
22. Hellenthal, G. *et al.* A genetic atlas of human admixture history. *Science* **343**, 747-751 (2014).
23. Seidman, D.N. *et al.* Rapid, Phase-free Detection of Long Identity-by-Descent Segments Enables Effective Relationship Classification. *Am J Hum Genet* **106**, 453-466 (2020).
24. Browning, S.R. & Browning, B.L. Accurate Non-parametric Estimation of Recent Effective Population Size from Segments of Identity by Descent. *Am J Hum Genet* **97**, 404-18 (2015).
25. Sticca, E.L., Belbin, G.M. & Gignoux, C.R. Current Developments in Detection of Identity-by-Descent Methods and Applications. *Front Genet* **12**, 722602 (2021).
26. Severson, A.L., Carmi, S. & Rosenberg, N.A. The Effect of Consanguinity on Between-Individual Identity-by-Descent Sharing. *Genetics* **212**, 305-316 (2019).
27. Browning, B.L. & Browning, S.R. Detecting identity by descent and estimating genotype error rates in sequence data. *Am J Hum Genet* **93**, 840-51 (2013).
28. Zhou, Y., Browning, S.R. & Browning, B.L. A Fast and Simple Method for Detecting Identity-by-Descent Segments in Large-Scale Data. *Am J Hum Genet* **106**, 426-437 (2020).
