# Supplementary material for: The Newfoundland and Labrador mosaic founder population descends from an Irish and British diaspora from 300 years ago
Source: Commun Biol. 2023 Apr 28;6:469. doi: 10.1038/s42003-023-04844-9 (PMC10147672; doi:10.1038/s42003-023-04844-9)
Supplement: Supplementary file 2 — Description of Additional Supplementary Files [file 42003_2023_4844_MOESM2_ESM.docx]

**Description of Additional Supplementary Files**

Listed below are the Supplementary Data associated with this Supplementary Information and Main Manuscript.

**Supplementary Data 1** - The demographics of NL *fineSTRUCTURE* clusters. For each cluster we show the breakdown of ancestry designations, and religious denomination.

**Supplementary Data 2** - The Hudson F_ST_ estimates with standard error between each of the NL *fineSTRUCTURE* cluster calculated from the admixtools2 R package.

**Supplementary Data 3** - The ancestry contribution estimates from each Irish or British cluster to each NL *finSTRUCTURE* cluster from *nnls*-based analysis of Identity-by-Descent segment sharing using IBD segments > 3 cM and < 15 cM.

**Supplementary Data 4** - The effective population (N_e_) estimates for NL *fineSTRUCTURE* clusters, and Irish and British regions using IBDNe.

**Supplementary Data 5** - The estimates of within-cluster Identity-by-Descent segment sharing in NL, Irish, and British clusters. We report the mean number of IBD segments and the mean total length of all IBD-segments >1 cM shared between the average pair of individuals in the same cluster. We also report the 95% confidence intervals.

**Supplementary Data 6** - The Runs-of-Homozygosity (ROH) estimates for NL, Irish, and British clusters. We report the average sum total of ROH > 1cM (with 95% confidence intervals), and ROH in four length bins, [4,8), [8,12), [12,20), and [20,300).

**Supplementary Data 7** - Measures of shared genetic drift between NL, and Ireland and England measured by the *qpDstat* program using the test *D*(YRI, NL, England, Ireland) where; YRI are Yorubans from the 1000 Genomes Phase 3 dataset, NL is any one NL *fineSTRUCTURE* cluster, England is individuals from the *S&W England* cluster, and Ireland is individuals with membership in either the *NW Munster* or *SE Leinster* clusters. We report the *D* estimate and the standard error calculated by *qpDstat*.

**Supplementary Data 8** - Measure of NL-specific drift with Patterson’s *D* statistic using *qpDstat*. We report the estimate and standard errors for the two tests *D*(YRI, X, England, NL), and *D*(YRI, X, Ireland, NL) where; YRI are Yorubans from the 1000 Genomes Phase 3 dataset, X is any one NL *fineSTRUCTURE* cluster, England is individuals from the *S&W England* cluster, Ireland is individuals with membership in either the *NW Munster* or *SE Leinster* clusters, and NL is 500 random NL individuals not members of the tested cluster X.
